# A synaptic signal for novelty processing in the hippocampus

**DOI:** 10.1038/s41467-022-31775-6

**Published:** 2022-07-15

**Authors:** Ruy Gómez-Ocádiz, Massimiliano Trippa, Chun-Lei Zhang, Lorenzo Posani, Simona Cocco, Rémi Monasson, Christoph Schmidt-Hieber

**Affiliations:** 1 Institut Pasteur, Université Paris Cité, Neural Circuits for Spatial Navigation and Memory, Department of Neuroscience, F-75015 Paris, France; 2 Sorbonne Université, Collège Doctoral, F-75005 Paris, France; 3 Laboratory of Physics of the École Normale Supérieure, PSL Research and CNRS UMR 8023, Sorbonne Université, Université Paris Cité, F-75005 Paris, France; 4 Present Address: Department of Neuroscience, Karolinska Institutet, 17177 Stockholm, Sweden; 5 Present Address: Center for Theoretical Neuroscience, Mortimer B. Zuckerman Mind Brain Behavior Institute, Columbia University, New York, NY USA

**Keywords:** Network models, Cellular neuroscience, Hippocampus, Neural circuits

## Abstract

Episodic memory formation and recall are complementary processes that rely on opposing neuronal computations in the hippocampus. How this conflict is resolved in hippocampal circuits is unclear. To address this question, we obtained in vivo whole-cell patch-clamp recordings from dentate gyrus granule cells in head-fixed mice trained to explore and distinguish between familiar and novel virtual environments. We find that granule cells consistently show a small transient depolarisation upon transition to a novel environment. This synaptic novelty signal is sensitive to local application of atropine, indicating that it depends on metabotropic acetylcholine receptors. A computational model suggests that the synaptic response to novelty may bias granule cell population activity, which can drive downstream attractor networks to a new state, favouring the switch from recall to new memory formation when faced with novelty. Such a novelty-driven switch may enable flexible encoding of new memories while preserving stable retrieval of familiar ones.

## Abstract (web summary)

Memory formation and recall are complementary processes within the hippocampus. Here the authors demonstrate a synaptic signal of novelty in the hippocampus and provide a computational framework for how such a novelty-driven switch may enable flexible encoding of new memories while preserving stable retrieval of familiar ones.

## Introduction

The hippocampus is essential for the formation and recall of episodic memories^1–4^. New memories need to be formed when the differences between ongoing experiences and previously stored representations exceed a threshold for behavioural relevance. Such novel memory formation requires a process of neuronal discrimination, whereby memory representations are encoded and stored in hippocampal cell assemblies that differ from those formed during previous experience. In turn, episodic memory recall is thought to result from the reactivation of previously stored patterns of neural activity, even when the inputs from upstream circuits are degraded or incomplete. Such neuronal generalisation is supported by attractor network dynamics arising from recurrent connectivity among hippocampal principal neurons in the CA regions, which enables the network to reinstate the activity of previously established neuronal assemblies^2,4–9^. However, memory formation and recall put conflicting requirements on hippocampal computations, as the reliable retrieval of familiar representations supported by robust attractor properties opposes the formation of new neuronal assemblies for the storage of novel episodic memories. How the hippocampal network reconciles these conflicting demands to achieve an optimal balance between memory formation and recall remains unclear^10–12^.

The dentate gyrus, situated immediately upstream of the CA3 region, appears well suited to solve this problem, as it performs neuronal discrimination by orthogonalising multimodal inputs from the entorhinal cortex through sparse firing activity and cellular expansion^2,6–8,12–21^. Hence, the dentate gyrus could be charged with the task of detecting novelty and selectively reporting it to downstream circuits, instructing them to store a new representation through a shift towards a different attractor state. However, experimental data have shown that the dentate gyrus robustly reports differences between any environments, independent of whether they are novel or familiar^12^.

Several requirements on the hippocampal memory system can explain that the dentate gyrus acts as a neutral difference detector. First, some aspects of a given experience might be categorised as familiar and thus lead to recall, while others are identified as novel and thereby favour encoding of a new memory. For example, when encountering a familiar location, it is equally important to retrieve the corresponding memory through generalisation as it is to detect the differences between the present episode and the memorised representation through discrimination to update memory with the ongoing experience^21–23^. Therefore, decorrelated outputs from the dentate gyrus need to be able to simultaneously support the recall of familiar representations and drive the formation of new representations in downstream regions. Moreover, whether a novel experience is sufficiently behaviourally relevant to merit encoding as a separate memory depends on the current behavioural context, general alertness, and arousal state, which are typically conveyed by extrahippocampal signals^22,24–26^. Given that the dentate gyrus performs neuronal discrimination steadily, regardless of whether the ongoing experience is novel or familiar, how can its robust discrimination code be flexibly recruited when a new memory needs to be formed?

To address this question, we obtained in vivo whole-cell patch-clamp recordings from dentate gyrus granule cells in head-fixed mice trained to explore and distinguish between familiar and novel virtual environments^12,27^. We report that granule cells consistently show a task-dependent small and transient depolarisation of their membrane potential when an animal encounters a novel environment. This depolarisation can be abolished by local application of atropine, indicating that it depends on metabotropic acetylcholine receptors. We then developed a simple but biologically plausible neural network model to investigate the computational consequences of the observed novelty-triggered depolarisation. The computational model suggests that the observed synaptic response to environmental novelty, leading to a bias in the granule cell population activity, could drive the CA3 attractor network to a new state, thereby favouring discrimination during memory encoding, as opposed to the default generalisation underlying recall when the animal navigates in a familiar environment. Thus, we propose a framework for how the hippocampus can effectively encode novel memories while preserving stable retrieval of familiar ones.

## Results

### Mice distinguish between familiar and novel virtual environments

**Fig. 1:**
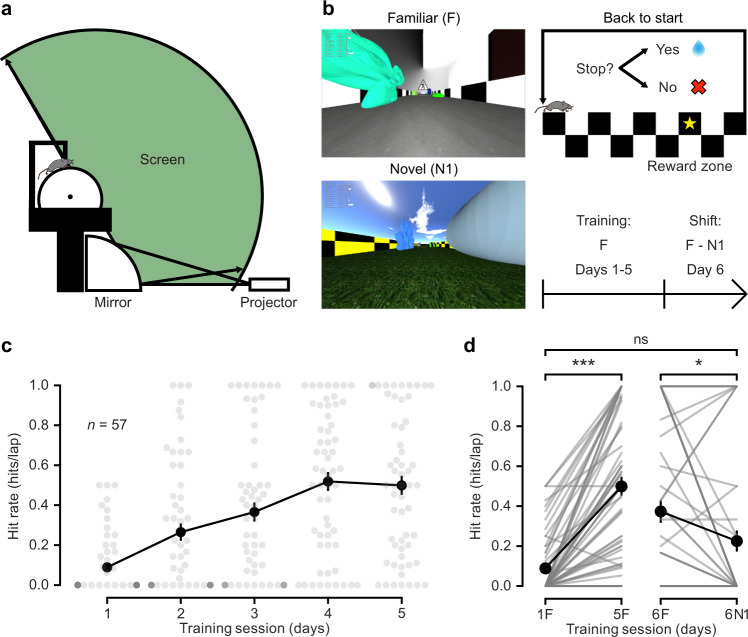
Mice distinguish between familiar and novel virtual environments. **a** Schematic of the virtual reality setup (adapted from Schmidt-Hieber & Häusser, 2013). **b** Left: view along the long axis of the corridor in the familiar (F, top) and the novel 1 (N1, bottom) virtual reality environments. Top right: schematic of the navigation task. Mice are trained to stop in a reward zone to obtain a reward. At the end of the corridor, mice are ‘teleported’ back to the beginning. Bottom right: behavioural protocol timeline. **c** Behavioural performance across training sessions, measured as hit rate (see “Methods”). Grey circles represent individual animals. Black circles represent the mean ± s.e.m. across all animals (*n* = 57 mice). **d** Left: comparison of behavioural performance during the first (1F) and the last (5F) training sessions in the familiar environment (hit rate: 0.09 ± 0.02 hits/lap and 0.50 ± 0.05 hits/lap, respectively; *n* = 57 mice, two-sided Wilcoxon signed-rank test, *T* = 7.0, Bonferroni-corrected *p* = 5 × 10^−9^). Right: comparison of behavioural performance in the familiar (6F) and in the novel environment 1 (6N1) during the 6th training session (hit rate: 0.37 ± 0.06 hits/lap and 0.23 ± 0.05 hits/lap, respectively; *n* = 57 mice, two-sided Wilcoxon signed-rank test, *T* = 43.0, Bonferroni-corrected *p* = 0.02; 1 F versus 6N1, two-sided Wilcoxon signed-rank test, *T* = 108.5, Bonferroni-corrected *p* = 0.09). Data are presented as the mean ± s.e.m. Source data are provided as a Source Data file.

To explore the behavioural effect of novelty, we used an immersive virtual reality setup adapted for rodent head-fixed navigation (Fig. 1a). We created three visually rich virtual reality environments with identical geometry and task logic but with different sets of proximal and distal cues^28^ and wall and floor textures. After habituation to the virtual reality setup, we trained water-restricted mice to navigate in the virtual corridor of the familiar environment (F) and stop at an un-cued reward zone for a defined period to obtain a water reward. Mice were ‘teleported’ back to the beginning upon arrival at the end of the corridor (Fig. 1b). We observed a marked increase in performance across five consecutive daily training sessions in the familiar environment (F) of 20–30 min each, as measured by licking in anticipation of reward delivery (hit rate: 0.09 ± 0.02 hits/lap during the 1st session and 0.50 ± 0.05 hits/lap during the 5th session; *n* = 57 mice, Wilcoxon signed-rank test, Bonferroni-corrected *p* < 0.001; Fig. 1c, d), reflecting the reliable learning of the task. In the sixth training session, we introduced the novel environment 1 (N1) and compared the performance in both environments. We found that while the performance in the familiar environment (F) reflected the learning of the task, the performance in the novel environment 1 (N1) decreased to a level comparable to untrained animals (hit rate: 0.37 ± 0.06 hits/lap in F and 0.23 ± 0.05 hits/lap in N1; *n* = 57 mice, Wilcoxon signed-rank test, Bonferroni-corrected *p* < 0.05; first session in F versus N1, Wilcoxon signed-rank test, Bonferroni-corrected *p* > 0.05; Fig. 1d). These results indicate that mice can rapidly learn to perform the virtual reality navigation task and that this learning is environment-specific, as evidenced by the ability of the animals to show different behaviour when exposed to novelty.

### Granule cells transiently depolarise in response to saliency

**Fig. 2:**
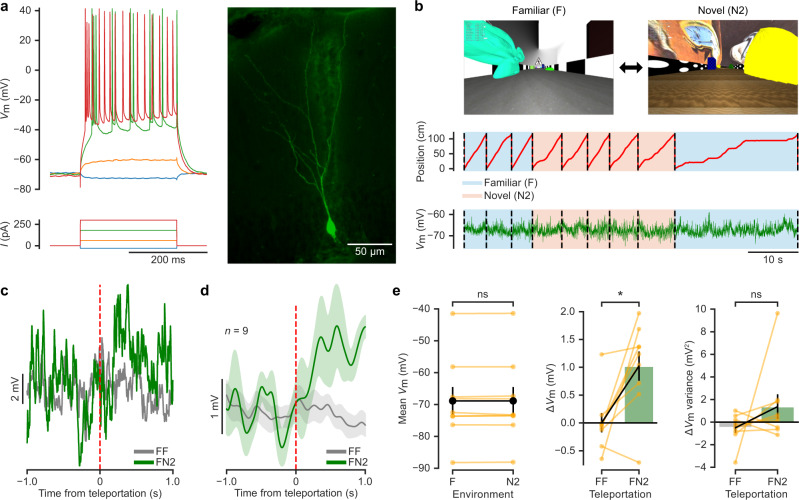
Granule cells transiently depolarise in response to saliency. **a** Example recording from a granule cell (from 73 independent recordings with similar results). Left: membrane potential (*V*_m_) responses to current (*I*) pulse injections. Right: fluorescence image obtained after biocytin filling during the recording. **b** Example recording during a behavioural discrimination experiment alternating between the familiar (F) and a novel environment (N2). Top: view along the long axis of the corridor in the familiar (F, left) and the novel 2 (N2, right) environments. Traces show animal position along the corridor (middle) and *V*_m_ (bottom). **c** Teleportation-aligned average from a representative recording. Traces represent the mean *V*_m_ aligned to teleportation events. The average from teleportations within the familiar environment (FF) is shown in grey and the single teleportation from the familiar to the novel environment (FN2) is shown in green. Teleportation time is indicated by the vertical red dashed line. **d** Teleportation-aligned average across multiple recordings. Traces represent the mean ± s.e.m. (*n* = 9 cells) of low-pass filtered *V*_m_ aligned to teleportation events. Teleportations within the familiar environment (FF) are shown in grey and teleportations from the familiar to the novel environment (FN2) are shown in green. Teleportation time is indicated by the vertical red dashed line. **e** Left: summary of mean *V*_m_ for the familiar (F) and the novel (N2) environments (−68.9 ± 4.3 and −68.9 ± 4.4 mV, respectively; *n* = 9 cells, two-sided Wilcoxon signed-rank test, *T* = 15.0, *p* = 0.4). Middle: ∆*V*_m_ summary for teleportations within the familiar environment (FF) and teleportations from the familiar to the novel environment (FN2) (0.00 ± 0.17 mV and 1.02 ± 0.26 mV, respectively; *n* = 9 cells, two-sided Wilcoxon signed-rank test, *T* = 2.0, *p* = 0.02). Right: ∆*V*_m_ variance summary for teleportations within the familiar environment (FF) and teleportations from the familiar to the novel environment (FN2) (−0.5 ± 0.4 mV^2^ and 1.4 ± 1.1 mV^2^, respectively; *n* = 9 cells, two-sided Wilcoxon signed-rank test, *T* = 13.0, *p* = 0.3). Data are presented as the mean ± s.e.m. Source data are provided as a Source Data file. See also Supplementary Fig. 1.

To study the synaptic integration processes underlying this behavioural discrimination^29,30^, we obtained whole-cell patch-clamp recordings from granule cells while mice performed the task, but this time alternating between laps in the familiar environment (F) and in another novel environment 2 (N2) to ensure that the animals had never encountered the novel environment before (Fig. 2a, b). We restricted our experiments to one recorded cell per animal and we confirmed the accuracy of the recording site in all cases using electrophysiological and anatomical criteria (see “Methods”). We then computed the teleportation-aligned average of the membrane potential traces around the teleportation events and compared the teleportations within the familiar environment (FF) and between the familiar environment and the novel environment 2 (FN2) (Fig. 2c–e). We did not observe any significant difference between the overall mean membrane potential recorded during the total time spent in the familiar and novel environment (−68.9 ± 4.3 mV in F and −68.9 ± 4.4 mV in N2; *n* = 9 cells, Wilcoxon signed-rank test, *p* > 0.05; Fig. 2e). However, we found a consistent membrane potential depolarisation following the teleportation event when the novel environment 2 (N2) was introduced for the first time (mean Δ*V*_m_ during 1 s after the teleportation: 1.02 ± 0.26 mV for FN2 teleportations versus 0.00 ± 0.17 mV for FF teleportations; *n* = 9 cells, Wilcoxon signed-rank test, *p* < 0.05; Fig. 2e and Supplementary Fig. 1a, b). This *V*_m_ depolarisation was not explained by a change in animal movement upon encountering the novel environment, as we did not observe a significant difference in Δspeed across teleportations (Δspeed: −0.8 ± 0.4 cm/s for FF, 0.0 ± 0.9 cm/s for FN2, −1.2 ± 1.3 cm/s for N2N2, −0.7 ± 1.9 cm/s for N2F; *n* = 9 cells, Friedman test, *p* > 0.05; Supplementary Fig. 1f). The depolarisation lasted for ~2 s (Supplementary Fig. 1c), was not accompanied by a change in membrane potential variance (1.4 ± 1.1 mV^2^ for FN2 teleportations versus −0.5 ± 0.4 mV^2^ for FF teleportations; *n* = 9 cells, Wilcoxon signed-rank test, *p* > 0.05; Fig. 2e) and its magnitude was not correlated with the mean membrane potential of the cells (FN2 Δ*V*_m_ versus mean *V*_m_: Pearson’s correlation coefficient, *r* = −0.19, *p* > 0.05; Supplementary Fig. 1d). Since these recordings were obtained using a blind unbiased approach, the consistent observation of a transient membrane potential depolarisation suggests that this synaptic phenomenon is not restricted to a specific subset of dentate gyrus neurons.

### A small depolarisation may increase the fraction of spiking cells

How does the observed synaptic response to novelty affect population activity in the dentate gyrus? To quantify this effect, we used a bootstrap procedure that accounts for the membrane potential dynamics observed in our dataset, the temporal dynamics of the subthreshold novelty signal, and the reported distribution of baseline membrane potentials as well as the action potential threshold of granule cells in vivo^27^ (see “Methods”). This strategy allowed us to estimate that during a 1 s time window after teleportation to novelty, the fraction of spiking neurons increases by ~30% (Supplementary Fig. 1g). This large relative increase can be explained by the highly sparse activity of granule cells in vivo^10,19,31^. Our result indicates that a relatively small membrane potential depolarisation observed at the individual neuron level, if driven by a generalised network mechanism, would entail the recruitment of a small but—in relative terms—sizable amount of silent neurons to the active population. While the depolarisation affects all granule cells equally, only those that are close to firing threshold will be recruited, providing specificity for cells that are synaptically activated upon transition to novelty. In addition, a small depolarisation will also increase the firing rates of the small population of active neurons, and it may drive some granule cells into firing bursts of action potentials, as has previously been described in vivo^27,32^. Such burst firing would further amplify the effect of the transient depolarisation on population activity.

### Isolated visual stimuli fail to depolarise granule cells

**Fig. 3:**
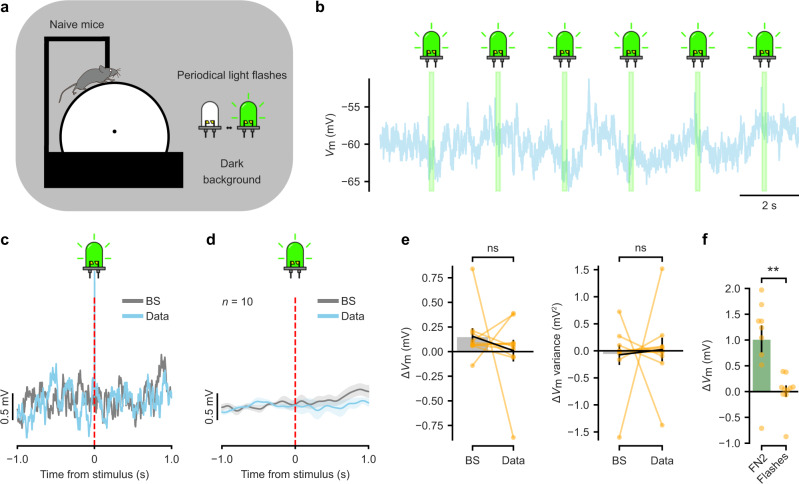
Isolated visual stimuli fail to depolarise granule cells. **a** Schematic of the experiment. Naive head-fixed mice move freely on a linear treadmill in a dark environment. Collimated LED light is flashed periodically on the eyes of the animals. **b** Example whole-cell recording from a granule cell during a light-flash experiment. The trace shows membrane potential (*V*_m_) and light flash events are indicated in green. **c** Stimulus-aligned average from a representative recording. The blue trace represents the mean *V*_m_ aligned to light flash events. The grey trace represents the mean *V*_m_ from a bootstrap (BS) dataset. Light flash event time is indicated by the vertical red dashed line (also note the presence of the stimulation artefact). **d** Stimulus-aligned average across multiple recordings. The blue trace represents the mean ± s.e.m. (*n* = 10 cells) of low-pass filtered *V*_m_ aligned to light flash events. The grey trace represents the mean ± s.e.m. of low-pass filtered *V*_m_ from a bootstrap dataset. Light flash event time is indicated by the vertical red dashed line. **e** Left: ∆*V*_m_ summary for the bootstrap dataset events (BS) and light flash events (Data) (0.16 ± 0.08 and 0.01 ± 0.11 mV, respectively; *n* = 10 cells, two-sided Wilcoxon signed-rank test, *T* = 21.0, *p* = 0.5). Right: ∆Vm variance summary for the bootstrap dataset events (BS) and light flash events (Data) (−0.07 ± 0.19 and 0.02 ± 0.22 mV^2^, respectively; *n* = 10 cells, two-sided Wilcoxon signed-rank test, *T* = 23.0, *p* = 0.7). **f** Bar graph showing the ∆*V*_m_ summary for teleportations from the familiar to the novel environment (FN2) and in response to light flashes (Flashes) (FN2, *n* = 9 cells versus Flashes, *n* = 10 cells; two-sided Mann–Whitney U test, *U* = 9.0, *p* = 0.001). Left bar: same data as in Fig. 2e middle, right bar. Right bar: same data as in e left, right bar. Data are presented as the mean ± s.e.m. Source data are provided as a Source Data file.

To probe if the observed synaptic response to saliency requires the animal’s engagement in the behavioural task, we presented isolated visual stimuli to untrained head-fixed mice moving freely on the treadmill surrounded by a uniformly dark screen (Fig. 3a). We then obtained whole-cell patch-clamp recordings from granule cells and presented periodical flashes of LED collimated light directed to the mice’s eyes (Fig. 3b). We computed the stimulus-aligned average within a 1 s window around the light flash presentation across multiple recordings and compared it to a bootstrap dataset (Fig. 3c, d). In this case, we did not observe a significant difference in membrane potential (Δ*V*_m_: 0.01 ± 0.11 mV for the data and 0.16 ± 0.08 mV for the bootstrap; *n* = 10 cells, Wilcoxon signed-rank test, *p* > 0.05; Fig. 3e) or in membrane potential variance (0.02 ± 0.22 mV^2^ for the data and −0.07 ± 0.19 mV^2^ for the bootstrap; *n* = 10 cells, Wilcoxon signed-rank test, *p* > 0.05; Fig. 3e) within this time window. This Δ*V*_m_ in response to light flashes was significantly different from the one observed during teleportations from the familiar to the novel environment (FN2) (FN2, *n* = 9 cells versus flashes, *n* = 10 cells; Mann–Whitney U test, *p* < 0.01; Fig. 3f). The absence of a transient depolarisation when isolated visual stimuli are presented suggests that saliency by itself is not sufficient to trigger the synaptic effect observed before. Instead, our finding indicates that the targeted locomotor engagement of the animal in the behavioural task is necessary for this form of synaptic novelty detection.

### Atropine abolishes the *V*_m_ response to saliency in granule cells

**Fig. 4:**
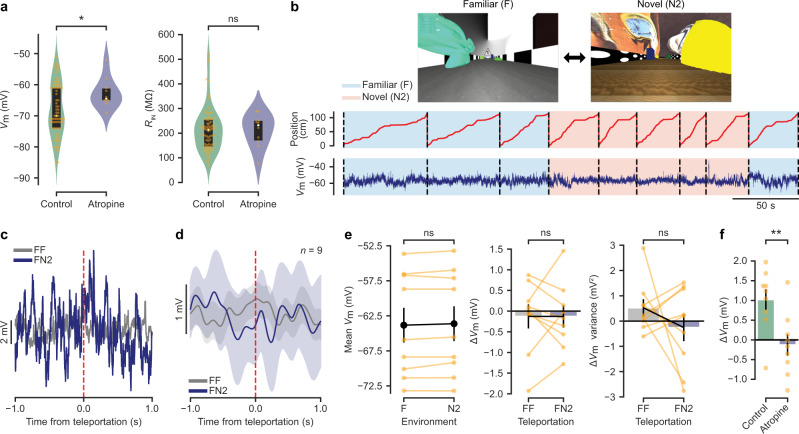
Atropine abolishes the *V*_m_ response to saliency in granule cells. **a** Left: membrane potential (*V*_m_) in granule cells under control conditions and after local injection of atropine (control: −68 ± 1 mV, median = −70 mV, 1st and 3rd quartiles = −74 and −61 mV, *n* = 73 cells; atropine: −63 ± 2 mV, median = −64.5 mV, 1st and 3rd quartiles = −65.0 and −61.3 mV, *n* = 10 cells; two-sided Mann–Whitney U test, *U* = 224.5, *p* = 0.03). Right: input resistance (*R*_IN_) in granule cells under control conditions and after local injection of atropine (control: 216 ± 11 MΩ, median = 214 MΩ, 1st and 3rd quartiles = 146 MΩ and 255 MΩ, *n* = 73 cells; atropine: 211 ± 21 MΩ, median = 232 MΩ, 1st and 3rd quartiles = 170 and 250 MΩ, *n* = 8 cells; two-sided Mann-Whitney U test, *U* = 331.0, *p* = 0.3). Box plots represent median and 1st and 3rd quartiles. **b** Example recording under atropine. Data presented as in Fig. 2b. **c** Teleportation-aligned average from a representative recording under atropine. Data presented as in Fig. 2c. **d** Teleportation-aligned average across multiple recordings under atropine (*n* = 9 cells). Data presented as in Fig. 2d. **e** Left: Summary of mean *V*_m_ for familiar (F) and novel (N2) environments after local injection of atropine (−63.8 ± 2.5 mV mV and −63.6 ± 2.5 mV, respectively; *n* = 9 cells, two-sided Wilcoxon signed-rank test, *T* = 12.0, *p* = 0.2). Middle: Summary of ∆*V*_m_ for teleportations within F (FF) and teleportations from F to N2 (FN2) (−0.13 ± 0.29 mV and −0.13 ± 0.27 mV, respectively; *n* = 9 cells, two-sided Wilcoxon signed-rank test, *T* = 22.0, *p* = 1.0). Right: ∆*V*_m_ variance summary for FF and FN2 teleportations (0.5 ± 0.3 mV^2^ and −0.3 ± 0.5 mV^2^, respectively; *n* = 9 cells, two-sided Wilcoxon signed-rank test, *T* = 16.0, *p* = 0.4). **f** Summary of ∆*V*_m_ for FN2 teleportations under control conditions and after local injection of atropine (control, *n* = 9 cells versus atropine, *n* = 9 cells; two-sided Mann–Whitney U test, *U* = 13.0, *p* = 0.009). Left bar: same data as in Fig. 2e middle, right bar. Right bar: same data as in e middle, right bar. Data are presented as the mean ± s.e.m. Source data are provided as a Source Data file. See also Supplementary Fig. 2.

As the neuromodulatory transmitter acetylcholine is thought to play a key role in orchestrating the network changes related to attention and task engagement in response to uncertainty^33–35^, we repeated our experiment while blocking metabotropic acetylcholine receptors using the non-specific muscarinic competitive antagonist atropine through local stereotaxic injection immediately prior to the recordings. We confirmed the accuracy of our drug application technique and the extent of diffusion of the injected volume by injecting a solution containing a fluorescent dye (BODIPY) in a subset of animals (Supplementary Fig. 2a; see “Methods”). In a separate group of animals, we applied atropine through chronically implanted cannulae prior to the behavioural discrimination session to confirm that local application of the drug does not impair task performance (Supplementary Fig. 2b–d; see “Methods”). Granule cells recorded after local application of atropine showed a more depolarised *V*_m_ baseline when compared with our previous recordings (atropine: −63 ± 2 mV, *n* = 10 cells; control: −68 ± 1 mV, *n* = 73 cells; Mann–Whitney U test, *p* < 0.05; Fig. 4a). Baseline input resistance did not differ between the two groups (atropine: 211 ± 21 MΩ, *n* = 10 cells; control: 216 ± 11 MΩ, *n* = 73 cells; Mann-Whitney U test, *p* > 0.05; Fig. 4a). We then performed teleportation-aligned average analysis as described above (Fig. 4b–e). As observed in the experiment without drug application, we did not detect a significant difference between the mean membrane potential recorded during the total time spent in both virtual environments (−63.8 ± 2.5 mV in F and −63.6 ± 2.5 mV in N2; *n* = 9 cells, Wilcoxon signed-rank test, *p* > 0.05; Fig. 4e). Notably, we did not find a change in membrane potential or membrane potential variance between FF and FN2 teleportations when analysing a 1 s time window after the teleportation events (Δ*V*_m_: −0.13 ± 0.27 mV for FN2 teleportations and −0.13 ± 0.29 mV for FF teleportations; *n* = 9 cells, Wilcoxon signed-rank test, *p* < 0.05. Δ*V*_m_ variance: −0.3 ± 0.5 mV^2^ for FN2 teleportations and 0.5 ± 0.3 mV^2^ for FF teleportations; *n* = 9 cells, Wilcoxon signed-rank test, *p* > 0.05; Fig. 4e). Moreover, the Δ*V*_m_ observed during FN2 teleportations after local injection of atropine was significantly different from the one observed in control animals (atropine: *n* = 9 cells; control: *n* = 9 cells; Mann–Whitney U test, *p* < 0.01; Fig. 4f). The absence of membrane potential depolarisation in response to novelty under muscarinic blockade suggests that this effect depends on metabotropic cholinergic signalling.

### Increased dentate gyrus activity may initiate a map switch in CA3

**Fig. 5:**
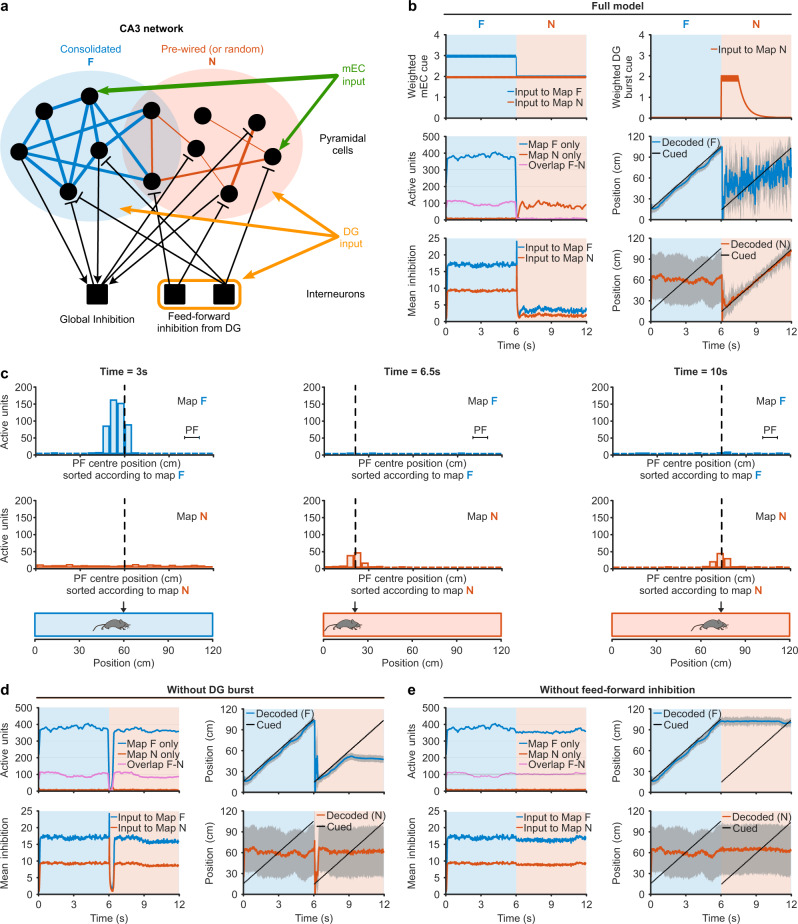
Increased dentate gyrus activity may initiate a map switch in CA3. **a** CA3 network model. Consolidated (blue) and weak (red) recurrent connections between cells (black dots) support, respectively, the familiar (F, blue) and novel (N, red) maps. Units receive inhibitory connections (black lines, ending with a bar) from interneurons (black squares), and excitatory inputs from medial entorhinal cortex (mEC, green) and dentate gyrus (DG, orange). Interneurons receive excitatory connections from CA3 cells (global inhibition, black lines ending with an arrow) and transient inputs from DG (feed-forward inhibition). **b** Top row: Dynamics of mEC (left) and transient DG (right) inputs to CA3 units. Middle and bottom rows: Network dynamics. (Middle left) Time traces of the number of active units in CA3. Neurons with a place field in only one of the two maps are plotted in blue (F map) or red (N map). Active units with a place field in both maps are represented in purple. (Bottom left) Total inhibitory inputs to CA3 units from global and feed-forward inhibition. (Right: middle and bottom) Comparison between cued (black) and decoded (average over place field centres positions of active units) positions for maps F (blue, middle) and N (red, bottom). Shaded areas represent the standard deviation associated with the decoded position. **c** Snapshots of the activity for three positions (bottom row): in F (left), right after teleportation (centre), and in N (right). Blue and red bars represent the number of active cells with a place field (PF) centre in the corresponding bin in, respectively, the F (first row) and N (second row) maps. Black dashed lines represent the position cued through the mEC input. **d**, **e** Network dynamics for incomplete models (similar to panel **b** middle and bottom rows). **d** Model without the novelty-driven excitatory input from DG. Network activity is restored after the teleportation with a bump that follows the position in the F map for a short time. **e** Model without the feed-forward inhibition from DG. The activity bump in the F map remains active after the teleportation and prevents the formation of a new bump in map N.

To investigate the potential downstream effects of the transient increase in granule cell activity, we implemented a network model of CA3, subject to inputs from the dentate gyrus and the medial entorhinal cortex (mEC) (Fig. 5a). The model is based on the continuous attractor theory of spatial memory and navigation^36^. Place cells in the CA3 model network are supported by excitatory inputs from mEC, in correspondence with the physical position of the rodent in a specific environment, and by a pattern of recurrent connections. We assume that repeated exposures to the familiar environment (F), through a learning process not explicitly modelled here, produce a pattern of connections forming a single consolidated map. In addition, we assume the existence of other connections that could support alternative representations^37^ and be used for a novel environment (N). The structure of connections in this sub-network can be either pre-wired or purely random (see “Methods”). In the pre-wired sub-network scenario, the extra connections are weak and irregular, but match the statistics of the novel environment: cells that receive inputs from mEC for the same position of the animal have higher probability to be connected; this scenario is compatible with the so-called pre-play hypothesis, and is plausible if the novel and familiar environments are similar, as suggested by the observation of submerged hippocampal networks able to modify spatial representations after optogenetic stimulation^38^. In the random sub-network scenario, connections are drawn uniformly at random between pairs of cells, irrespectively of the statistics of their inputs from mEC; this scenario is expected to be realistic if the novel environment is entirely different from the familiar one.

Random projections from the dentate gyrus to CA3 neurons excite a fraction of CA3 neurons and contain no spatial selectivity (baseline inputs, Fig. 5b). Following ‘teleportation,’ this fraction of excited neurons transiently increases (Fig. 5b), and forms the support of the N-subnetwork. Furthermore, in our model, the dentate gyrus recruits feed-forward inhibition in a frequency-dependent manner^39,40^.

In the familiar (F) environment, place cells are activated by mEC inputs and the strong recurrent connections in the network supporting map F. This connectivity results in a large bump of activity, coding at all times for the position of the animal as it moves along the track (see Fig. 5b, middle right and 5c, left). Note that even if the rodent is in the familiar environment, mEC inputs excite some place cells which are shared with the immature ‘pre-map’ N (purple line in Fig. 5b, middle left), but are not sufficient to trigger substantial activity there (Fig. 5b, bottom right), as the global inhibition of the CA3 network allows for only one activity bump at a time.

Following teleportation to the novel environment, two phenomena take place as a result of the burst of activity in the dentate gyrus. On the one hand, activity in the CA3 network is temporarily strongly inhibited by feedforward inputs^41^ (Fig. 5b, bottom left), abolishing the activity bump in map F (Fig. 5c, middle). On the other hand, the enhanced inputs from dentate gyrus towards the sub-network supporting the new pre-map N (Fig. 5b, top right) randomly stimulate additional cells, allowing for the emergence of another collective state of activity.

In the pre-wired sub-network scenario this boost in activity initiates a coherent, self-sustained new bump of activity around the neurons receiving most inputs (Fig. 5c, middle). This new neural activity bump then persists after dentate gyrus inputs return to their baseline value, and robustly codes for the animal’s position in the novel map at all times (Fig. 5c, right), even though the inputs from mEC in the novel map are not stronger than the ones pointing towards the familiar map (Fig. 5b, top left).

In the random sub-network scenario, transient increase of inputs from the dentate gyrus and spatially selective inputs from the mEC combine to activate a small but statistically significant fraction of cells (Supplementary Fig. 5). As the animal explores the environment N for the first time this level of activity is maintained, even after the dentate gyrus inputs return to their baseline level, and the population of active cells changes in accordance with the motion of the animal. However, once the animal is teleported back to the origin of the track and initiates the second lap this pre-map N activity is unstable, as no extra inputs from dentate gyrus are now received by the CA3 network and the random pattern of recurrent connections may not be sufficient to sustain the active population of cells. Depending on the neural noise, the activity may either stay in pre-map N or switch back to map F (Supplementary Fig. 6b, left). However, we find that this instability disappears, and pre-map N persists when Hebbian learning of the connections is present (Supplementary Fig. 6b, right). As the connections of the initially random network strengthen, the pre-map N gets stabilised, and a position-dependent activity is present for all laps in the novel environment.

In both connectivity scenarios, the two-fold effects of the increase in activity in the dentate gyrus are crucial for the coherent switch from map F to pre-map N to take place (see Supplementary Movie). If no burst of excitatory inputs from the dentate gyrus to pre-map N accompanies the transition, the bump in map F is reinstated after its transient disappearance as the animal navigates in the novel environment (Fig. 5d). Furthermore, in the absence of transient inhibition in CA3, the bump of activity in map F is not erased and persists in the novel environment (Fig. 5e). Thus, the model shows that transient inputs from the dentate gyrus are essential to switch from a robust familiar representation to a weakly pre-formed novel map in the downstream attractor network. In addition, we have shown that this result is robust against the hypothesis about the (unknown) state of connectivity supporting the new map. If the connections of the sub-network supporting the pre-map N are statistically consistent with the spatial variations of the inputs from mEC in the novel environment, the bump of activity triggered by the increase of activity of the dentate gyrus during the first lap is stable and observed in subsequent laps when the dentate gyrus inputs are back to baseline level. Conversely, for random connectivity, consolidation of the connections (e.g. through a Hebbian-like learning mechanism) seems to be necessary to stabilise the novel map. This stabilisation takes place after a few explorations of the novel environment.

## Discussion

Here we show that dentate gyrus granule cells exhibit a transient small depolarisation of their membrane potential when animals are ‘teleported’ into a novel virtual environment. By contrast, isolated visual stimuli that are presented independently of the animal’s behaviour fail to evoke any systematic synaptic response. The observed novelty-dependent depolarisation can be blocked by local application of atropine, indicating that it depends on activation of muscarinic (metabotropic) acetylcholine receptors. While the amplitude of the depolarisation is only on the order of 1 mV, because of the sparse activity levels in the dentate gyrus we estimate that even such a small depolarisation can have a large effect on the relative increase in the proportion of firing neurons. Computational modelling reveals that this increased activity level in the dentate gyrus can push the downstream CA3 network from retrieving an existing representation of a familiar environment to forming a new one of the novel environment.

To support ongoing behaviour in an environment with mixed novel and familiar components, hippocampal circuits need to be able to store relevant novel information without disrupting the recall of familiar memories^21–23^. It has been suggested that upon transition into a novel environment, robustly decorrelated inputs from the dentate gyrus push downstream circuits to form a new neuronal representation (i.e. ‘global remapping’). However, the attractor dynamics of the CA3 circuit counteract this process by stabilising the familiar representation as the default mode^2,8,20,21^, thereby putting the two processes at odds with each other. Additional inputs containing information about the saliency and the unexpectedness of the novel environment^33^ may provide the required arbiter signal that decides whether the CA3 circuit forms a new neuronal representation or recalls a stored memory.

How is this ‘novelty signal’ conveyed to the hippocampal network? In contrast to the multimodal cortical inputs to the hippocampus, which carry information about distinct memory elements, subcortical inputs are known to contain information about ‘global’ internal brain states, such as attention, uncertainty, and arousal^24,25,33^. Signals of this sort could be transmitted to the hippocampus in the form of a generalised short-lived network perturbation that could in turn alter the manner in which information is processed in the circuit. This kind of global transitory change in circuit dynamics can occur in response to diverse neuromodulatory transmitters, which are crucial evolutionary-conserved elements for the reshaping and repurposing of neural circuits during associative and non-associative learning^25,42,43^. Different neuromodulatory systems appear to play distinct roles during mnemonic processing and, among these, cholinergic neuromodulation has been suggested to be the one in charge of signalling ‘absolute’ novelty to the hippocampus^22,44^. Indeed, theoretical models have suggested that increased cholinergic neuromodulation promotes encoding of novel information in the hippocampal network while reduced cholinergic neuromodulation favours consolidation of previously encoded patterns^34,44,45^. This notion is supported by experimental evidence of a rise in acetylcholine levels in the hippocampus when an animal encounters a novel spatial environment^46–49^ and of memory impairment by pharmacological blockade of cholinergic neurotransmission^50,51^, which selectively affects encoding while sparing retrieval^52^.

Previous studies have shown that optogenetic stimulation of cholinergic septohippocampal projections can recruit excitatory inputs to granule cells as a component of a bimodal synaptic response^53^. The inhibitory late component of this response is mediated by multiple intermediaries, including astrocytes and local interneurons, depends on nicotinic acetylcholine and GABA_A_ receptor channels, and can be revealed at a negative chloride reversal potential (~–88 mV) when septohippocampal fibres are synchronously stimulated at high frequencies. In our recordings, which were obtained at a physiological *E*_Cl_ for mature granule cells (~−72 mV)^54,55^, the excitatory component of the response appears to be isolated, as hyperpolarising inhibition may be absent due to the small driving force for chloride ions, low-frequency asynchronous activation of cholinergic inputs in vivo, and differences in synaptic transmission dynamics, such as baseline GABAergic tone, in awake versus anaesthetised animals^56,57^. Thus, while the transient short-lived signal that we observe is depolarising, potential shunting inhibition appearing at a later phase, depending on glial intermediaries, may explain why an increased cholinergic tone can lead to reduced overall granule cell activity in novel environments under some conditions^10,58^.

The depolarisation that we observe is sensitive to local application of atropine, suggesting that it depends on metabotropic acetylcholine receptors, which could also explain the seconds-long temporal dynamics of the signal. A role for these receptors in mediating the novelty signal that we observe is further supported by the aforementioned pharmacological studies, which affect memory encoding by specifically blocking muscarinic receptors^50–52^. From the five subtypes of muscarinic receptors, M_1_, M_2_, and M_4_ are the most widely expressed in the hippocampus^59^. Among these, the M_2_ subtype is expressed only in interneurons, while the M_1_ subtype is preferentially expressed in the somatodendritic compartment of principal cells^60,61^ and is known to enhance postsynaptic excitability and NMDA receptor activity by inhibiting potassium channels^62,63^ and calcium-activated SK channels^64,65^. These features make it a candidate driver of the membrane potential depolarisation that we observe in granule cells^35,50,51^, in accordance with the disappearance of the effect observed in our experiments when these receptors are blocked with atropine. This notion is also in line with recent studies showing that muscarinic acetylcholine receptors selectively drive the depolarisation and increase in bursting activity of principal cells in the hippocampus observed under conditions of high cholinergic tone^66^. In addition, other dentate gyrus cell types might also be involved in novelty processing: it has recently been reported that hilar mossy cells—which are also known to be targets of cholinergic neuromodulation^67,68^—play a key role during novelty detection in the hippocampus^69^, which raises the possibility that the synaptic response to novelty that we recorded in granule cells is not driven by the direct effect of acetylcholine on granule cells but instead indirectly through cholinergic modulation of local interactions in the intrahippocampal circuit. Genetically targeted manipulation of specific nicotinic and muscarinic acetylcholine receptors in combination with cell-specific neuronal recordings during novelty-associated behaviour will be necessary to clarify the precise mechanisms whereby acetylcholine modifies the synaptic properties of the hippocampal circuit in response to saliency.

How does the synaptic novelty signal affect the activity of individual granule cells? The small depolarisation that we observe will selectively affect the firing of neurons that are already synaptically activated: it will increase the firing rate of neurons that are actively spiking, and it will drive silent cells to fire spikes if their membrane potential is close to the action potential threshold. It has been shown that evoking spikes in previously silent granule cells in vivo at specific locations in the environment can lead to the induction of place fields, especially under novelty^70^. We, therefore, expect that at least part of the newly recruited active neurons will permanently fire upon transition into the environment that led to their activation when the animal first encountered it, even as the environment grows familiar. Such a process may facilitate the robust retrieval of the corresponding attractor state in CA3 over time.

Studies on the activity of granule cell populations have reported both decreased and increased activity as an animal familiarises itself with a novel environment over the time course of several minutes and more, and the long-term dynamics of this process remains to be delineated^10,58,69^. However, how the population activity changes at the moment when the animal transitions into a novel environment is unclear. As the overall activity levels in the dentate gyrus are notably sparse, the transient depolarisation will only recruit a small absolute number of neurons into the spiking population, and may only lead to firing of few or even single additional action potentials. Population recording techniques that are typically employed in virtual reality, such as 2-photon imaging^10,12^, are likely to miss these additional few spikes that occur only in a small number of neurons during a short time window^71^. Highly sensitive electrophysiological recordings with high temporal and single-spike resolution from large populations of neurons during instantaneous environment switches in virtual reality will be required to observe the predicted population response^72^.

How can a transient increase in dentate gyrus activity affect downstream circuits? By implementing a computational model of CA3, we reveal that a small and short excitation from the dentate gyrus, accompanied by transient increased inhibition^41^, can initiate a weak but self-sustained activity bump that encodes the rodent position in the representation (map) of the novel environment. Both the transient fast inhibition and the slower excitation required in this model can result from increased activity in the dentate gyrus, as mossy fibres provide both monosynaptic excitation as well as disynaptic feedforward inhibition to CA3 pyramidal cells. The excitation-inhibition balance is governed by complex frequency-dependent dynamics, with net inhibition predominating at low presynaptic firing frequencies and a switch to net excitation occurring as frequency increases^39,40^. Thus, the ability to invert the polarity of the mossy fibre-CA3 pyramidal neuron synapse in a frequency-dependent manner may be necessary when switching between information processing modes in the hippocampal circuit under the regulation of extrahippocampal signals. Moreover, mossy fibre-CA3 synapses show pronounced post-tetanic potentiation in response to natural bursting activity patterns described in vivo^27,32,73^, which could be triggered by the small depolarisation that we observe. This form of synaptic plasticity would further boost excitatory drive, supporting the new activity bump.

Our model suggests that the mechanism described above for map switching is largely robust against the initial connectivity structure that will ultimately support the cell assembly coding for the novel environment. In one scenario we have assumed the existence of a pre-configured subnetwork of cells, partially coherent with the correlations of activity in the novel environment. The existence of pre-configured assemblies is at the basis of the so-called preplay phenomenon^74^, and is compatible with the observed emergence of alternative maps during silencing of place-cell assemblies^37^. Assuming pre-wired connectivity is also plausible if the novel and the familiar environments share many features, as could be the case in the setup considered here. Notably, contrary to previous models of switching between two equally consolidated maps^75^, switching to an immature map crucially requires transient inhibition, in agreement with reports that somatic inhibition is transiently increased following novelty in CA1^41,76^. In another, opposite scenario we have considered the case of a naive, purely random connectivity network. Our simulations show that, under the dual inhibitory/excitatory action of the dentate gyrus, a weak activity bump, mostly driven by mEC inputs can still be formed in a CA3 cell assembly that differs from the one representing the familiar environment. This bump persists during the first lap, but is unstable in subsequent laps due to the lack of coherence with the naive recurrent connectivity. We have shown that the bump could, however, be stabilised through Hebbian-like plasticity mechanisms. Though not indispensable it is likely that learning processes, possibly involving reconfiguration of inhibitory circuits^77^, would ultimately strengthen and reshape the primitive cell assembly even when pre-configured representations are assumed.

In our experimental data, we do not only observe a transient depolarisation during teleportations from familiar to novel environments, but also during the first teleportations between novel environments (N-N), and to a smaller extent upon teleportation from the novel back to the familiar environment (N-F; Supplementary Fig. 1b). How would these signals affect neuronal dynamics in the model? In both scenarios (N-N and N-F), the transient excitatory signal from the dentate gyrus would further facilitate the activation of the proper representation for the current environment, as inputs from recurrent collaterals, mEC and DG would align to shift network activity towards the correct map. However, this additional DG-driven input is not necessary when the environment has already been explored at least once, as we have shown for subsequent teleportations to a partially consolidated novel map (Supplementary Fig. 6). These results suggest that the transient depolarisation during the first transition from F to N is needed as a substitute for mEC and recurrent inputs, which should be weak for a novel environment. Recurrent excitatory inputs can then be rapidly strengthened by Hebbian learning, reducing the need for a DG-driven excitatory signal for the teleportations after the first one. For the same reason a transition to a familiar environment does not require additional excitatory inputs from the DG: the strong mEC cues (consolidated with learning) activate units in the correct cell assembly (F) which, in turn, activate a stable bump of activity in the F map due to the positive feedback given by the recurrent, consolidated connections, as we have previously observed^75^.

We propose a framework for how cholinergic modulation of the dentate gyrus can affect network dynamics downstream in area CA3. Based on our computational model, we speculate that the synaptic novelty signal in the dentate gyrus switches attractor networks from generalisation to discrimination. Future experimental work will have to test this hypothesis more directly by showing that specific inhibition of the synaptic novelty signal in the dentate gyrus disrupts the formation of novel representations in downstream networks, and consequently storage of new memories. Conversely, weak transient activation of a large population of granule cells may lead to the formation of new assemblies in CA3 even in the absence of any environmental changes.

What could be the benefit of separating the location of the cholinergic modulation in the dentate gyrus from its downstream effect, instead of directly activating CA3 neurons? One advantage is that only those granule cells that are synaptically activated during transition to novelty, bringing them close to or above the firing threshold, are specifically modulated. As the dentate gyrus produces codes that strongly discriminate between familiar and novel environments, only a subset of inputs to CA3 that is highly specific for the novel environment increases its activity. A novelty signal that non-specifically acted on CA3 neurons would activate, among others, assemblies coding for the familiar environment, as their activity is maintained by their attractor properties, even if the animal is already in a novel environment. This stable attractor state would then prevent novel representations from forming in CA3. Another advantage is that a transient increase in the fraction of active neurons enhances the separability of representations^78^. The depolarisation that we observe in the dentate gyrus in response to novelty could thus temporarily increase the discriminative power of the network, further favouring the establishment of a new neuronal assembly in CA3. Furthermore, activating inputs from the dentate gyrus to CA3 that are active or close to spiking threshold during teleportation to novel environments may induce Hebbian plasticity at specific dentate gyrus-to-CA3 synapses, so that novel information is rapidly stored in specific assemblies. The upstream cholinergic processes in the dentate gyrus that we describe here are not in conflict with previously reported cholinergic modulation of CA3;^1^ on the contrary, we suggest that they synergistically promote plasticity.

**Fig. 6:**
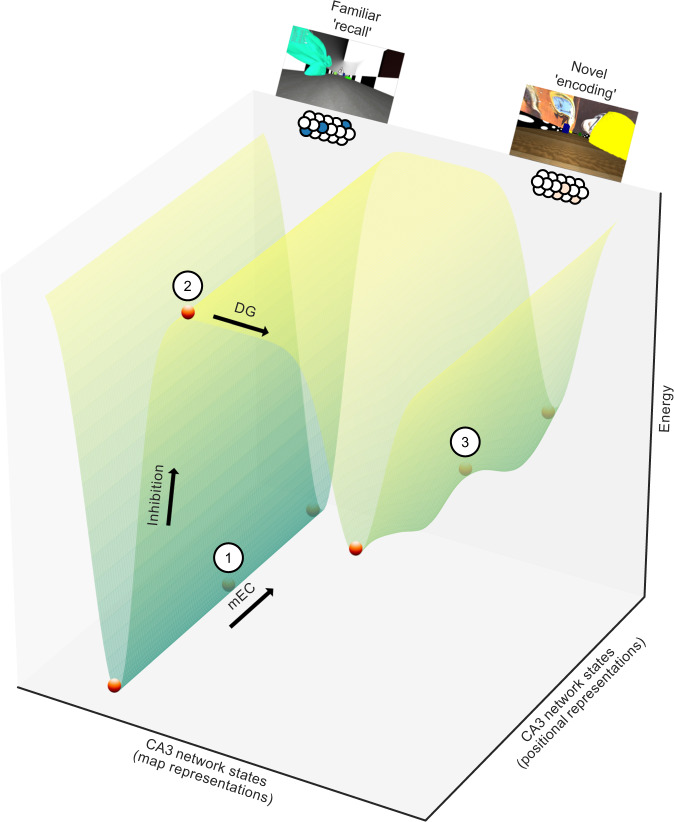
Switching between familiar and novel attractor states: proposed model. Energy landscape of CA3 network states representing different maps and different positions along the track (schematic). In a familiar environment, the CA3 network falls into an attractor state that is governed by strengthened recurrent synaptic connectivity, thereby performing generalisation during memory recall of familiar events. We propose that a small bias in the inputs from the dentate gyrus (DG) first recruits feed-forward inhibition, thereby lifting the network state out of the deep trough representing the familiar environment. Direct excitation from the dentate gyrus then pushes the CA3 network into a different attractor state with initially weaker, pre-existing recurrent connectivity, thereby performing discrimination during novelty encoding. Numbers represent network states corresponding to the model snapshots shown in Fig. 5c (1: Fig. 5c, left; 2: Fig. 5c, middle; 3: Fig. 5c, right). mEC, medial entorhinal cortex.

Neuronal generalisation and discrimination are processes that must occur in concert, as familiar memories need to be robustly retrieved but also updated with relevant novel information. In addition, any experience consists of multiple elements in different physical dimensions that may need to be stored as separate memories depending on their behavioural relevance^21–23^. Inducing a small and transient universal ‘bias’ in the population code of the dentate gyrus when faced with novelty may provide a solution to this challenge, as the overall structure of the dentate gyrus population code is only weakly and briefly affected while the novel environment is learnt and familiarised. Novelty can thereby flexibly tag different dimensions of an experience to produce multifaceted memory representations (Fig. 6).

## Methods

### Animals and surgical procedures

All procedures were conducted in accordance with European and French regulations on the ethical use of laboratory animals for experimentation (EU Directive 2010/63/EU) and were reviewed and approved by the Ethics Committee of the Institut Pasteur CETEA (APAFIS#7771-2016112516084126 v1). 5–16 week-old male C57BL/6J wild-type mice (Janvier Labs) were used. Animals were housed in groups of four in polycarbonate individually ventilated cages equipped with running wheels and were kept under constant temperature and humidity with a 12 h inverted light/dark cycle and *ad libitum* access to food and water. All animals were treated identically. Multimodal analgesia (buprenorphine 0.05 mg/kg + meloxicam 5 mg/kg) was administered through intraperitoneal injection at least 30 min before any surgical intervention and the skin on the surgical area was infiltrated with lidocaine prior to incision. Antisepsis of the incision sites was performed with povidone-iodine (Betadine). Animals were anaesthetised with isoflurane (3% for induction, 1–2% for maintenance, vol/vol) and placed in a stereotaxic apparatus (Kopf Instruments). The corneal surfaces were protected from desiccation by applying artificial tear ointment and the body temperature was kept constant throughout the surgical intervention with a heating pad. The skin was incised with scissors and the periosteum was mechanically removed using a surgical bone scraper. Stainless-steel headposts (Luigs & Neumann) were attached to the animals’ skulls using dental cement (Super-Bond C&B, Sun Medical). Postoperative analgesia (meloxicam 5 mg/kg) was administered orally in combination with surgical recovery feeding gel (ClearH_2_O). Animals were allowed to recover from surgery for at least seven days preceding the start of the training sessions.

### Behavioural tasks in virtual reality

Three custom virtual reality environments were developed using the Blender Game Engine (http://www.blender.org) in conjunction with the Blender Python Application Programming Interface. All environments consisted of a 1.2 m-long linear corridor visually enriched with proximal and distal cues and floor and wall textures. The reward delivery trigger zone was placed in an un-cued location in the third quarter of the corridor to avoid proximity to either virtual boundaries or the teleportation trigger point and was identical in all environments. The warped environments were projected onto a spherical dome screen (⌀ 120 cm) using a quarter-sphere mirror (⌀ 45 cm) placed underneath the mouse, as described previously^79–81^. The screen covered ~240°, which corresponds to nearly the entire horizontal field of view of the mouse. Animals were head-fixed and placed on an air-supported polystyrene rolling cylinder (⌀ 20 cm) that they used as a treadmill to navigate the virtual scene. Cylinder rotation associated with animal locomotion was read out with a computer mouse (Logitech G500) and linearly converted to one-dimensional movement along the virtual reality corridor. Animals were extensively handled and habituated to the virtual reality setup before the onset of experimental procedures. Animals underwent 5 training sessions of 20–30 min each on consecutive days prior to the electrophysiological recordings. All training sessions were conducted during the dark phase of the light cycle of the mice. During the training period and the experiments, animals were water-restricted to 80% of their baseline weight to maximise their behavioural drive^82^. Body weight and general health status were monitored daily. Animals were trained to navigate the virtual corridor in the familiar environment (F) and to retrieve an 8% sucrose solution reward (10 μL) by stopping for at least 3 s in an un-cued reward zone placed at a fixed location of the corridor. Licking behaviour was monitored using a custom-made Arduino piezoelectric sensor coupled to the reward delivery spout. Animals were ‘teleported’ back to the beginning of the track upon crossing of a defined threshold near the end of the virtual corridor. As the virtual environments, training protocol and reward contingencies used in this study are different from the ones used in previous work^12^, the hit rate results are not directly comparable. After having completed the five-day training protocol in the familiar environment, a behavioural recording session was conducted in which laps in the familiar environment (F) were alternated with laps in the novel environment 1 (N1). The same environment alternation strategy was used during the electrophysiological recordings, using the familiar environment (F) and the novel environment 2 (N2). A purely behavioural session was conducted separately from the electrophysiological recordings since the latter are typically very short and therefore do not provide enough behavioural data to accomplish an accurate assessment of task performance.

### In vivo whole-cell patch-clamp recordings

Two craniotomies (⌀ ~0.5 mm) were drilled 3–24 h before the recording session for the recording electrode (right hemisphere, 2.0 mm caudal, and 1.5 mm lateral from Bregma) and the reference electrode (left hemisphere, 2.0 mm caudal and −1.5 mm lateral from Bregma). The *dura mater* was removed using fine forceps and the cortical surface was kept covered with artificial cerebrospinal fluid of the following composition (in mmol/L): 150 NaCl, 2.5 KCl, 10 HEPES, 2 CaCl_2_, 1 MgCl_2_, with pH adjusted to 7.2. In a subset of animals, 600 nL of 1 mmol/L atropine solution was injected with a large-tip glass micropipette at a depth of 1.7 mm from the cortical surface to selectively target the dentate gyrus^83^. The local injection of the drug was made through a standard patch-clamp pipette (thus being equivalent to the first patch-clamp pipette penetration in the ‘control’ group) and the anaesthetic and surgical procedures performed on the animals in the ‘control’ group and in the group in which atropine was locally applied prior to recordings were identical. Recording electrodes were pulled from filamented borosilicate glass (Sutter Instrument) and filled with internal solution of the following composition (in mmol/L): 135 potassium methanesulfonate, 7 KCl, 0.3 MgCl_2_, 10 HEPES, 0.1 EGTA, 3.0 Na_2_ATP, 0.3 NaGTP, 1 sodium phosphocreatine and 5 mg/mL biocytin, with pH adjusted to 7.2 with KOH. Osmolarity was 285–290 mOsm/L. All chemicals were purchased from Sigma. Pipette tip resistance was 4–8 MΩ. Electrodes were arranged to penetrate the brain tissue perpendicularly to the cortical surface at the centre of the craniotomy and the depth of the recorded cell was estimated from the distance advanced with the micromanipulator (Luigs & Neuman), taking as a reference the point where the recording electrode made contact with the cortical surface. Whole-cell patch-clamp recordings were obtained using a standard blind-patch approach, as previously described^12,27,84^. Only recordings with a seal resistance > 1 GΩ were included in the analysis. Recordings were obtained in current-clamp mode with no holding current. No correction was applied for the liquid junction potential. *V*_m_ signals were low-pass filtered at 10 kHz and acquired at 50 kHz. After completion of a recording, the patch recording electrode was gently withdrawn to obtain an outside-out patch to verify the integrity of the seal and ensure the quality of the biocytin filling. To synchronise behavioural and electrophysiological recordings, TTL pulses were triggered by the virtual reality system whenever a new frame was displayed (frame rate: 100 Hz) and recorded with both the behavioural and the electrophysiological acquisition systems.

### Implantation of cannulae for drug infusion in the dentate gyrus

To assess the effects of local application of atropine during behavioural experiments in a subset of animals (Supplementary Fig. 2b–d) under comparable conditions as during the behavioural control experiments (Fig. 1), stainless steel guide cannulae (gauge 26; P1 Technologies) were implanted bilaterally above the dentate gyrus (coordinates for craniotomy: from Bregma, anteroposterior +1.6–2.1 mm, mediolateral ±1.8–2.3 mm, angled at 25–28° towards medial from vertical; dorsoventral, 0.8–1.0 mm), anchored to the skull with dental cement (Super-Bond C&B, Sun Medical), and covered with dummy cannulae to reduce the risk of infection. Mice were allowed 3 weeks of recovery after this procedure before the start of the training sessions, with their health and general well-being assessed daily. Prior to the behavioural discrimination experiment, an infusion cannula (gauge 33; P1 Technologies) connected to a 1 µL Hamilton syringe via polyethylene tubing was inserted through each guide cannula, protruding 1.5 mm, to target the dentate gyrus. 350–400 nL of saline vehicle or 1 mM atropine in saline solution was infused bilaterally at a rate of 100 nL/min using a motorised pump (Legato 100, KD Scientific). To allow for even distribution of the infused volume, the injector was maintained in position for 3 min after the end of the infusion. Upon completion of the bilateral infusion procedure, mice were allowed to recover in their home cages for 45–60 min before proceeding to the behavioural experiment. To assess the location and extent of the infusions, a similar volume of the fluorophore BODIPY TMR-X (Invitrogen; 5 mM in PBS 0.1 M, DMSO 40%) was infused into the dentate gyrus through the cannulae. After 3 h, animals were deeply anaesthetised, and brains fixed through intracardiac perfusion with 4% paraformaldehyde solution (see Histology and microscopy). 60 µm-thick slices were prepared from the infused hippocampi. Slices were counterstained with DAPI and imaged using a spinning-disc confocal microscope (Opterra, Bruker). Mice were considered for further analysis if BODIPY fluorescence signals could be confirmed in the dentate gyrus (Supplementary Fig. 2b).

### Histology and microscopy

Immediately upon completion of a successful recording, animals were deeply anaesthetised with an overdose of ketamine/xylazine administered intraperitoneally and promptly perfused transcardially with 1× phosphate-buffered saline followed by 4% paraformaldehyde solution. Brains were extracted and kept immersed overnight in 4% paraformaldehyde solution. 60–70 μm-thick coronal slices were prepared from the recorded hippocampi. Slices were stained with Alexa Fluor 488–streptavidin to reveal biocytin-filled neurons and patch electrode tracts. DAPI was applied as a nuclear stain to reveal the general anatomy of the preparation. Fluorescence images were acquired using a spinning-disc confocal microscope (Opterra, Bruker) and analysed using ImageJ. The accuracy of the recording coordinates was confirmed in all cases by identification of either the recorded neuron or the recording electrode tract.

### Estimation of the population effect of the synaptic novelty signal

We devised a bootstrap method to estimate the increase in spiking probability caused by the synaptic novelty signal. We selected *n* = 4 membrane potential recordings from silent neurons obtained in the familiar environment based on the stability of the membrane potential and its variance throughout the recordings. We then generated a list of *n* = 1000 baseline membrane potential values, drawn from a distribution representing the known characteristics of granule cell membrane potentials in vivo^27^: we used a skewed Gaussian distribution with the mean and standard deviation set to the published values (–73 ± 9 mV, mean ± s.d.). The skewness (4) was set to match the published minimum and maximum values that would be expected in a subset of *n* = 26 recordings (~–90 to ~–50 mV). We shifted each of the *n* = 4 membrane potential recordings 1000 times so that their baseline membrane potentials (mean membrane potential during the first second of the recording) matched the list of the generated baseline membrane potential values. For each of these shifted membrane potential traces, we added the depolarisation waveform that we observed during transitions from familiar to novel environments sampling point by sampling point, and estimated whether the membrane potential trace crossed the action potential threshold during the 1 s period following the sampling point, either in the presence or in the absence of the novelty signal. The threshold was adopted from the same dataset as the baseline membrane potential values^27^. This strategy allows us to compute the fraction of neurons that would be recruited into the firing population if the novelty transition had taken place at this sampling point. We thereby take into consideration the known action potential threshold and distribution of membrane potentials, the variance and dynamics of membrane potential in vivo, and the dynamics of the novelty signal.

### CA3 network model: neural representations and recurrent connectivity

An auto-associative neural network of $n= \,20,000$ binary (0,1) units was implemented as a model of CA3. Place fields (PF) were randomly assigned to 20% of the $n$ units in the familiar environment F, uniformly centred on $p=400$ regularly spaced points along the track (PF diameter = 33 pts). The distance between two adjacent points represents 0.3 cm to reproduce the physical length of the track (120 cm) in the experiment (PF diameter = 9.9 cm). A neural representation of the F environment was stored in the network (Eq. 1) by associating a specific pattern of activity $\xi$ to each position on the track. To do so, the activity pattern ${\xi }^{\mu }$ ($\mu =1,2,\ldots ,{p}$) was generated by randomly choosing 330 units (as in Guzman et al. (2016)^9^, resulting in sparsity $a=0.0165$ in our network) among those with a PF overlapping the position considered. This procedure yielded correlated activity patterns (corresponding to memory representations) at spatially close positions.

#### Coupling matrix for map F

The coupling matrix ${J}^{(F)}$ was defined through the clipped Hebb rule,

1 $${J}_{{ij}}^{(F)}=\,{{\min }}\left(1,{\sum }_{\upmu =1}^{p}{\xi }_{i}^{{{\upmu }}}{\xi }_{j}^{{{\upmu }}}\right).$$

Such couplings carve a quasi-continuous attractor model of the environment^36^.

#### Coupling matrix for pre-map N

In the random coupling scenario (Supplementary Figs. 5 and 6), the matrix ${J}_{{ij}}^{(N)}$ connected only the units in the N cell assembly ($0.2{n}$ randomly chosen units) not overlapping with the set of F units in Fig. 5a. To each cell $i$ in this subset was assigned a normally distributed random number of input connections (mean ${\mu }^{J}=20,$ standard deviation ${\sigma }^{J}=3,$ values rounded to the closest integer) from other cells in the same set. The selected connections were then set to ${J}_{{ij}}^{(N)}=1,$ all the couplings from the other units $j$ were instead set to 0. In the pre-wired scenario, couplings ${J}_{{ij}}^{(N)}$ supporting the pre-map N were defined in the same way as for map F, based on another random subset of $0.2{n}$ place cells. Furthermore, couplings were multiplicatively shrunk by random factors < 1 (beta-distribution, parameters $\alpha =0.7 \, < \, 1,\beta =1.2 \, > \, 1$). As a result, all connections were reduced in strength, and many were close to zero (Supplementary Fig. 3). The beta distribution was chosen as its two parameters allowed us (a) to have many couplings close to zero, thus making the pre-map N not only weaker but also sparser than map F, (b) to have, at the same time, some large couplings with intensities similar to the ones in map F. The similar behaviours of the model under both scenarios (random coupling and pre-wired) suggest that the particular choice of the distribution may not be essential for the proper functioning of the network.

#### Full coupling matrix

The excitatory synaptic matrix $J$ for the CA3 network was defined as

2 $${J}_{{ij}}={C}_{{ij}}\,\times \,\left({J}_{{ij}}^{(F)}+{J}_{{ij}}^{(N)}\right)$$

where the connectivity matrix ${C}_{{ij}}={{{{\mathrm{0,1}}}}}$ randomly assigned 1200 input connections $j$ to each unit $i$, in agreement with estimates of the connectivity^9^.

### CA3 network model: network dynamics

Place-cells’ activities ${s}_{i}={{{{\mathrm{0,1}}}}}$ were updated from time $t$ to $t+1$ according to the following probability:

3 $$P({s}_{i}\left(t+1\right)=1)= \;	{{{{{\rm{L}}}}}}\Big({{h}_{i}^{{{{{{\rm{RC}}}}}}}(t)-{g}_{i}\,S(t)+h}_{i}^{{{{{{\rm{mEC}}}}}}}(t)+{h}_{i}^{{{{{{{\rm{DG}}}}}}}\mbox{-}{{{{{\rm{base}}}}}}}(t)\\ 	+ \;{h}_{i}^{{{{{{\rm{DG}}}}}}\mbox{-}{{{{{\rm{excit}}}}}}}(t){-h}_{i}^{{{{{{\rm{DG}}}}}}\mbox{-}{{{{{\rm{inhib}}}}}}}(t)\Big)$$

with complementary probability $1-P$ for the update ${s}_{i}(t+1)=0$. Here, $i$ is the index of the unit, and ${{{{{\rm{L}}}}}}$ is the sigmoidal function ${{{{{\rm{L}}}}}}(h)=\frac{1}{\pi }{{{\tan }}}^{-1}\left(\frac{h-G}{T}\right)+\frac{1}{2}$, with noise parameter $T=0.1$, in agreement with the order of magnitude estimated for similar network models^85^, and activation threshold $G=2.31$^9^. The argument of ${{{{{\rm{L}}}}}}$ is the sum of the global inhibition with five inputs, that we now describe.

#### Recurrent inputs from CA3

Place cells receive inputs through CA3 recurrent couplings from other pyramidal cells, ${h}_{i}^{{RC}}(t)={\sum }_{j=1}^{n}{J}_{{ij}}{s}_{j}(t)$, where the connectivity matrix $J$ is described above. The presence of interneurons produces a global inhibition component proportional to the total network activity $S(t)={{\max }}({S}_{{\min }},{\sum }_{j=1}^{n}{s}_{j}(t)).$ Here, we choose ${S}_{{\min }}=50$ neurons to ensure that this global inhibition has a minimal nonzero value counterbalancing the DG baseline input (see below). The proportionality factor is ${g}_{i}^{F}=0.035$ for units $i$ contributing to the memory of the F map and ${g}_{i}^{N}=0.015$ for units involved in the N pre-map only. The differential contribution of global inhibition to units in the F and N sub-networks mirrors the different strengths of excitatory synaptic input from recurrent collaterals in the two maps. Complex patterns of synaptic plasticity have been reported in populations of CA1 and CA3 interneurons^86,87^, suggesting that learning and consolidation of new representations in excitatory synapses could be stabilised and regulated by a simultaneous strengthening of synapses with inhibitory interneurons.

#### Inputs from mEC

Inputs from the medial entorhinal cortex (mEC) to CA3 are spatially selective, acting on 50% of the place cells, chosen at random, among those involved in the activity pattern ${\xi }^{\mu }$associated with the current position of a virtual rodent. To account for consolidation of map F, input intensities on cells coding for environment F are stronger than for environment N while the rodent is navigating environment F. Conversely, while navigating in environment N, mEC inputs of equal strengths are sent to the two maps. At each time step the mEC input was cued to the network according to the following equation:

4 $${h}_{i}^{{{{{{\rm{mEC}}}}}}}(t)={\sum }_{{{{{{\rm{Map}}}}}}=\{F,N\}}{A}_{i}^{{{{{{\rm{mEC}}}}}},\,{{{{{\rm{Map}}}}}}}(t)\,{\delta }_{i}^{{{{{{\rm{mEC}}}}}},\,{{{{{\rm{Map}}}}}}}{{e}}^{-\left(t-{t}^{{{{{{\rm{mEC}}}}}}}\right)/{\tau }^{{{{{{\rm{mEC}}}}}}}}$$

where ${A}_{i}^{{{{{{{\rm{mEC}}}}}}},{{{{{{\rm{Map}}}}}}}}$ is the strength of the input to unit $i$, dependent both on the map which unit $i$ contributes to and on the environment the virtual rodent is in, ${\delta }_{i}^{{{{{{\rm{mEC}}}}}},{{{{{\rm{Map}}}}}}}$ indicates if unit $i$ receives the input associated to one of the two maps, ${t}^{{{{{{\rm{mEC}}}}}}}$ is the time step at which the cue was last updated and ${\tau }^{{{{{{\rm{mEC}}}}}}}$ is the timescale of decay of the cue. An update of this cue coincides with an update of the rodent’s position occurring once every 5 time steps. When the cue is updated the set of units receiving the input is drawn as follows: ${\delta }_{i}^{{{{{{\rm{mEC}}}}}},{{{{{\rm{Map}}}}}}}=1$ for $\frac{{a\; n}}{2}$ random CA3 units $i$ that contribute to the activity pattern ${\xi }^{\mu }$representing the rodent’s position in the map considered at time $t$ and 0 for the other units. The other parameters entering Eq. (4) are: ${A}_{i}^{{{{{{\rm{mEC}}}}}},{F}}=3$ for map F units during navigation of environment F, ${A}_{i}^{{{{{{\rm{mEC}}}}}},{F}}=2$ while in environment N, ${A}_{i}^{{{{{{\rm{mEC}}}}}},N}=2$ for pre-map N units (for navigation in both environments) and ${\tau }^{{{{{{\rm{mEC}}}}}}}=100$.

#### Inputs from the dentate gyrus

CA3 receives three types of inputs from the dentate gyrus in our model: baseline (*DG-base*), excitatory (*DG-excit*) and inhibitory (*DG-inhib*). *DG-base* inputs target a random 2% fraction of all CA3 units at all times, with no spatial or map selectivity. *DG-excit* inputs are transiently activated upon entrance in a novel environment only, and act on a 2% fraction of randomly chosen units in the sub-network supporting pre-map N, with the same intensity as mEC inputs in the novel environment. *DG-inhib* inputs excite interneurons in CA3, effectively modelling a short-term mechanism of feed-forward inhibition to CA3 linked to the transient increase of DG activity upon novelty. The three corresponding contributions appearing in Eq. (3) have the same formal expression as in Eq. (4), with the following parameters:

- ${A}^{{{{{{{\rm{DG}}}}}}}\mbox{-}{{{{{{\rm{base}}}}}}}}={A}^{{{{{{{\rm{DG}}}}}}}\mbox{-}{{{{{{\rm{excit}}}}}}}}=2$ for all cells and all maps (note however that cells not included in the N sub-network would not receive the *DG-excit* cue, see ${\delta }_{i}^{{{{{{{\rm{DG}}}}}}}\mbox{-}{{{{{{\rm{excit}}}}}}}}$). ${A}_{i}^{{{{{{{\rm{DG}}}}}}}\mbox{-}{{{{{{\rm{inhib}}}}}}}}=0.016\times {N}_{{{{{{\rm{inter}}}}}}}$, where ${N}_{{{{{{\rm{inter}}}}}}}$ is the number of interneurons place-cell $i$ is connected to. We choose this number to be Poisson distributed, with mean $500\times {{{{\mathrm{5000}}}}}/{{{{\mathrm{20,000}}}}}=125$, which corresponds to the case of a set of 5000 interneurons (20% of the CA3 population), each connected to 500 randomly chosen place cells;
- $${\tau }^{{{{{{{\rm{DG}}}}}}}\mbox{-}{{{{{{\rm{base}}}}}}}}={\tau }^{{{{{{{\rm{DG}}}}}}}\mbox{-}{{{{{{\rm{excit}}}}}}}}={30,\tau }^{{{{{{{\rm{DG}}}}}}}\mbox{-}{{{{{{\rm{inhib}}}}}}}}=3$$;
- ${\delta }_{i}^{{{{{{{\rm{DG}}}}}}}\mbox{-}{{{{{{\rm{base}}}}}}}}=1$ for 2% of all CA3 units chosen at random and ${\delta }_{i}^{{{{{{{\rm{DG}}}}}}}\mbox{-}{{{{{{\rm{base}}}}}}}}=0$ otherwise. For this cue the summation over maps is removed from Eq. (4).
- ${\delta }_{i}^{{{{{{{\rm{DG}}}}}}}\mbox{-}{{{{{{\rm{excit}}}}}}},{{{{{{\rm{Map}}}}}}}}=0$ for all units $i$ when the rodent is in environment F. ${\delta }_{i}^{{{{{{{\rm{DG}}}}}}}\mbox{-}{{{{{{\rm{excit}}}}}}},{N}}=1$ for $2 \%$ of the units in the N sub-network, chosen at random while the rodent is in map N. This value corresponds to a 20% increase of DG input in the model, less than predicted from the experimental data (~30%) to ensure that our prediction is robust. In addition, ${\delta }_{i}^{{{{{{{\rm{DG}}}}}}}\mbox{-}{{{{{{\rm{inhib}}}}}}}}=1$ for all place cells.

*DG-base* cues are updated once every 5 time steps. Here, again, this update of the cue corresponds to a new random set of units being drawn to receive this input (i.e., ${\delta }_{i}^{{{{{{{\rm{DG}}}}}}}\mbox{-}{{{{{{\rm{base}}}}}}}}$). *DG-excit* inputs are sent to CA3 units starting from the teleportation time and updated 14 times (once every 5 time steps, cue sent for 15 times in total). The set of units receiving the *DG-excit* input was uniformly drawn for each update of the cue according to the rule reported for ${\delta }_{i}^{{{{{{{\rm{DG}}}}}}}\mbox{-}{{{{{{\rm{excit}}}}}}}}$. The *DG-inhib* inputs are cued to the network only once at the time of teleportation, either to the same or to a different environment.

Alternative sets of parameters (i.e., strength of global inhibition and fraction of units involved in each map) were also tested to check the stability of the model (Supplementary Fig. 4). To account for the lower excitatory synaptic input in simulations with random unstructured connectivity (Supplementary Figs. 5 and 6), some parameters were slightly tuned: the fraction of units targeted by the DG baseline input was changed from 2 to $5 \%$, the strength of DG burst input was raised to ${A}^{{{{{{{\rm{DG}}}}}}}\mbox{-}{{{{{{\rm{excit}}}}}}}}=3$ and, finally, the coefficient of global inhibition for units in map N was lowered to ${g}_{i}^{N}=0.01$. In all figures, time steps were scaled by a factor of 20 ms to match the average speed of the rodent along the track.

### CA3 network model: Hebbian learning

The experimental data suggest that the transient increase in DG activity is largest during the first transition from a familiar to a novel environment, suggesting that the stability of the N map cannot rely on this input during subsequent teleportations in the novel environment (N-N). Synaptic plasticity in CA3 may support subsequent activations of the novel representation while forming and consolidating the novel map. To test this hypothesis, we implemented the following learning rule^88–90^ to model synaptic plasticity in the CA3 network:

5 $${J}_{{ij}}^{(t+1)}={{{{{\rm{max }}}}}}\left(0,{{{{{\rm{min }}}}}}\left(1,\,{J}_{{ij}}^{(t)}+\eta {C}_{{ij}}\,{\sigma }_{i}^{{{{{{{\rm{post}}}}}}}}(t)\,{\sigma }_{j}^{{{{{{{\rm{pre}}}}}}}}(t)\right)\right)$$

where the post- and presynaptic functions ${\sigma }_{i}(t)$ are defined as follows:

6 $${\sigma }_{i}^{{{{{{{\rm{post}}}}}}}}\left(t\right)=\frac{{h}_{i}^{{{{{{{\rm{BCM}}}}}}}}\left(t\right)}{{\varPhi }_{i}\left(t\right)}\left({h}_{i}^{{{{{{{\rm{BCM}}}}}}}}\left(t\right)-{\varPhi }_{i}\left(t\right)\right)$$

7 $${\sigma }_{i}^{{{{{{{\rm{pre}}}}}}}}\left(t\right)={s}_{i}\left(t\right)-\frac{1}{{\tau }^{{{{{{{\rm{learn}}}}}}}}}{\sum }_{{t}^{{\prime} }=1}^{{\tau }^{{{{{{{\rm{learn}}}}}}}}}{s}_{i}\left(t-{t}^{{\prime} }\right)$$

8 $${\varPhi }_{i}\left(t\right)=\frac{c}{{\tau }^{{{{{{{\rm{BCM}}}}}}}}}{\sum }_{{t}^{{\prime} }=0}^{{\tau }^{{{{{{{\rm{BCM}}}}}}}}}\,{\left[{h}_{i}^{{{{{{{\rm{BCM}}}}}}}}\left(t-{t}^{{\prime} }\right)\right]}^{2}$$

9 $${h}_{i}^{{{{{{{\rm{BCM}}}}}}}}\left(t\right)={{{{{\rm{L}}}}}}\left({h}_{i}^{{{{{{{\rm{RC}}}}}}}}\left(t\right)\right)$$

The parameters used were $\eta =0.1,$ ${\tau }^{{{{{{{\rm{learn}}}}}}}}=22,$ ${\tau }^{{{{{{{\rm{BCM}}}}}}}}=400,$ $c=9.5$ and $T=0.1$. This learning rule was applied once every 2 time steps when the virtual rodent was in environment N, assuming that the F map is already fully consolidated. Supplementary Fig. 6 shows the effect of learning in the case of an initially unstructured synaptic connectivity supporting the formation of the novel map.

### Data analysis and statistics

To analyse subthreshold membrane potential, *V*_m_ traces were digitally low-pass filtered at 5 kHz and resampled at 10 kHz. *V*_m_ traces were subsequently high-pass filtered at >10^–5^ Hz to remove slow trends such as reference drifts. Action potentials were removed from the traces by thresholding to determine action potential times and then replacing 2 ms before and 10–20 ms (depending on the action potential shape) after the action potential peak with an interpolated straight line. Data are presented as the mean ± s.e.m., unless stated otherwise. Statistical significance was assessed using two-sided Mann–Whitney U tests for unpaired data and two-sided Wilcoxon signed-rank tests for paired data. Multiple comparisons in dependent samples were initially evaluated using Friedman tests and the *p* values for single comparisons corrected according to the Bonferroni method. Indications of statistical significance correspond to the following values: ns *p* > 0.05, * *p* < 0.05, ** *p* < 0.01, *** *p* < 0.001. All analyses were carried out using custom-made Python scripts.

### Previous use of the data in other work

Some of the recordings included in the present study have also been used for previous work^12^, where the specificity of subthreshold responses for the familiar and novel environments was analysed.

### Reporting summary

Further information on research design is available in the Nature Research Reporting Summary linked to this article.

## Supplementary information

Supplementary Information

Description of Additional Supplementary Files

Supplementary Movie 1

Supplementary Code

Reporting Summary

## Data Availability

A source data file supporting the findings of this study is provided with this paper and its supplementary information files. Additional binary data files are available from the corresponding author upon request. Source data are provided with this paper.

## Source data

Source Data

## Code availability

MATLAB code supporting the network model is provided with this paper and its supplementary information files.
