# Supplementary material for: A synaptic signal for novelty processing in the hippocampus
Source: Nat Commun. 2022 Jul 15;13:4122. doi: 10.1038/s41467-022-31775-6 (PMC9287442; doi:10.1038/s41467-022-31775-6)
Supplement: Supplementary file 1 — Supplementary Information [file 41467_2022_31775_MOESM1_ESM.pdf]

A synaptic signal for novelty processing in the hippocampus

Ruy Gómez-Ocádiz[1,2,3], Massimiliano Trippa[4], Chun-Lei Zhang[1], Lorenzo Posani[1,5], Simona Cocco[4], Rémi Monasson[4], Christoph Schmidt-Hieber[1]✉

1 Institut Pasteur, Université Paris Cité, Neural Circuits for Spatial Navigation and Memory, Department of Neuroscience, F-75015 Paris, France
2 Sorbonne Université, Collège Doctoral, F-75005 Paris, France
3 Present address: Department of Neuroscience, Karolinska Institutet, 17177 Stockholm, Sweden
4 Laboratory of Physics of the École Normale Supérieure, PSL Research and CNRS UMR 8023, Sorbonne Université, Université de Paris, F-75005 Paris, France
5 Present address: Center for Theoretical Neuroscience, Mortimer B. Zuckerman Mind Brain Behavior Institute, Columbia University, New York, NY, USA
✉ Correspondence: christoph.schmidt-hieber@pasteur.fr

Supplementary Information

Supplementary Fig. 1

Supplementary Fig. 2

Supplementary Fig. 3

Supplementary Fig. 4

Supplementary Fig. 5

Supplementary Fig. 6

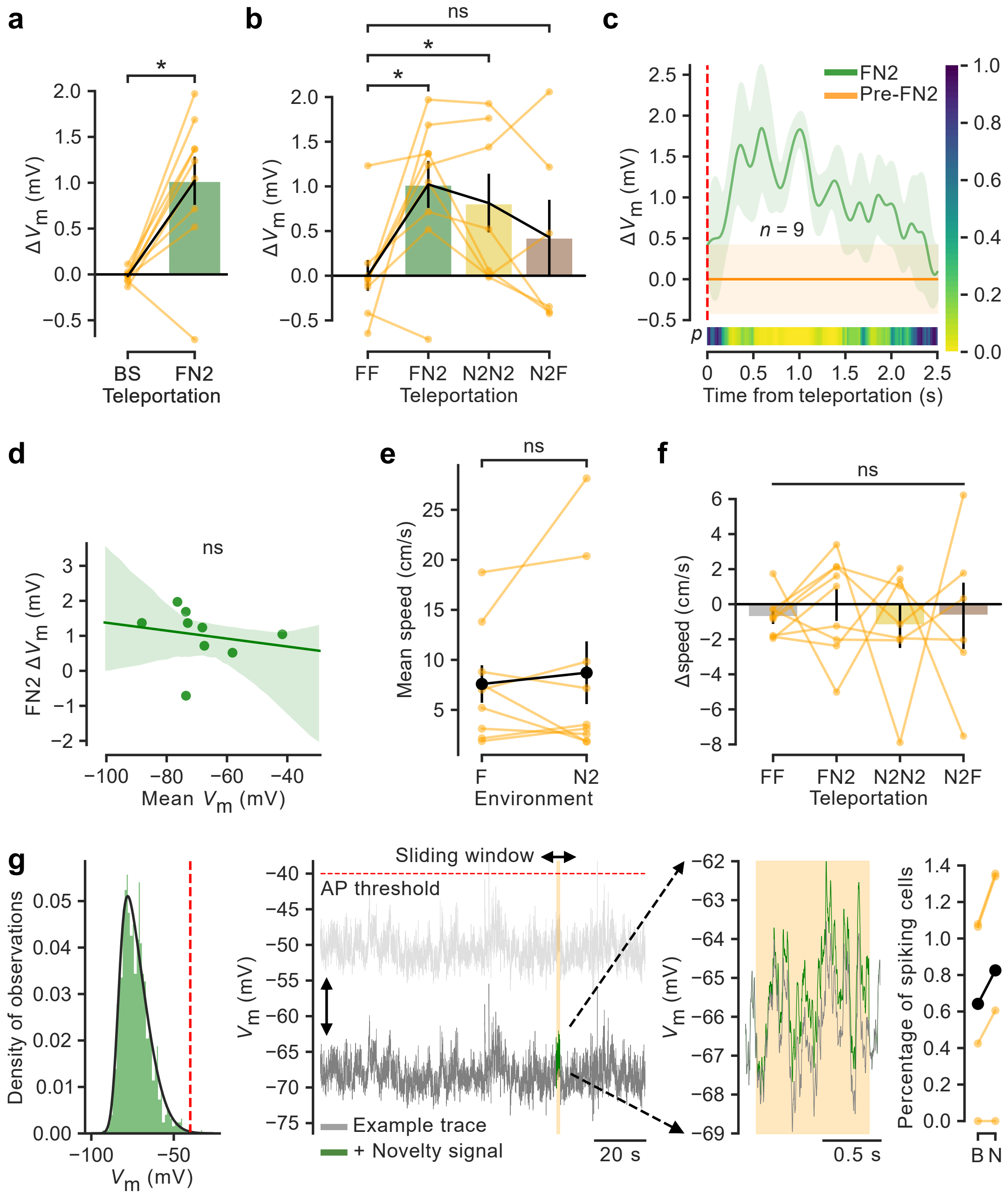

Supplementary Fig. 1 Supplementary analysis to Fig. 2. a ΔV_m summary for FN2 teleportations tested against a bootstrap obtained from the same dataset (Bootstrap: -0.02 ± 0.02 mV, FN2: 1.02 ± 0.26 mV; $n = 9$ cells, two-sided Wilcoxon signed-rank test, $T = 2.0$, $p = 0.02$). Right bar: same data as in Fig. 2e middle, right bar. **b** ΔV_m summary for all teleportations (0.00 ± 0.17 mV for FF, 1.02 ± 0.26 mV for FN2, 0.81 ± 0.33 mV for N2N2, 0.43 ± 0.42 mV for N2F; $n = 9$ cells; FF versus FN2, two-sided Wilcoxon signed-rank test, $T = 2.0$, Bonferroni-corrected $p = 0.046$; FF versus N2N2, two-sided Wilcoxon signed-rank test, $T = 1.0$, Bonferroni-corrected $p = 0.03$; FF versus N2F, two-sided Wilcoxon signed-rank test, $T = 6.0$, Bonferroni-corrected $p = 0.2$). **c** Teleportation-aligned average across multiple recordings showing the temporal dynamics of the subthreshold depolarisation in response to novelty. The green trace represents the mean ± s.e.m. ($n = 9$ cells) of the low-pass filtered ΔV_m recorded 2.5 s after the FN2 teleportation events. The orange trace represents the average mean ± s.e.m. of a 2.5 s period preceding the teleportation event. Teleportation time is indicated by the vertical red dashed line. The heat map represents p values for the difference between traces in 1 ms time windows (two-sided Wilcoxon signed-rank test). **d** Correlation between Mean V_m and FN2 ΔV_m ($n = 9$ cells, Pearson's correlation coefficient, $r = -0.19$, $p = 0.6$). **e** Summary of mean speed for the familiar (F) and the novel (N2) environments (7.6 ± 1.9 cm/s and 8.7 ± 3.1 cm/s, respectively; $n = 9$ cells, two-sided Wilcoxon signed-rank test, $T = 21.0$, $p = 0.9$). **f** Δspeed summary during 1 s after the teleportation event for all teleportations (-0.8 ± 0.4 cm/s for FF, 0.0 ± 0.9 cm/s for FN2, -1.2 ± 1.3 cm/s for N2N2, -0.7 ± 1.9 cm/s for N2F; $n = 9$ cells, Friedman test, $df = 3$, $Q = 1.8$, $p = 0.6$). **g** Bootstrap method to estimate the increase in percentage of spiking granule cells caused by the synaptic novelty signal. Left: histogram of $n = 1000$ synthetic baseline V_m values drawn from a skewed Gaussian distribution (black line) representing the known characteristics of granule cell membrane potentials *in vivo*[27] (see Methods). The action potential (AP) threshold (red dashed line) was adopted from the same dataset as the baseline V_m values. Middle: example V_m trace (grey) from a recording of a silent neuron that we obtained in the familiar environment. The trace is shifted 1000 times to match its baseline V_m (mean V_m during the first second of the recording) to the list of baseline V_m values drawn from the distribution. The orange shaded area represents a sliding window during which the depolarisation waveform observed during transitions from familiar to novel environments was added. The green trace represents the corresponding segment of the example trace after addition of the depolarisation waveform. Right: percentage of spiking cells in the absence (bootstrap, B) and in the presence (novelty, N) of the depolarisation waveform observed during the 1-second transition period from familiar to novel environments, calculated for $n = 4$ V_m traces from recordings of silent neurons obtained in the familiar environment (bootstrap [B]: 0.64%; novelty [N]: 0.83%; relative increase: 28.6%). V_m, membrane potential. Data are presented as the mean ± s.e.m.. Source data are provided as a Source Data file.

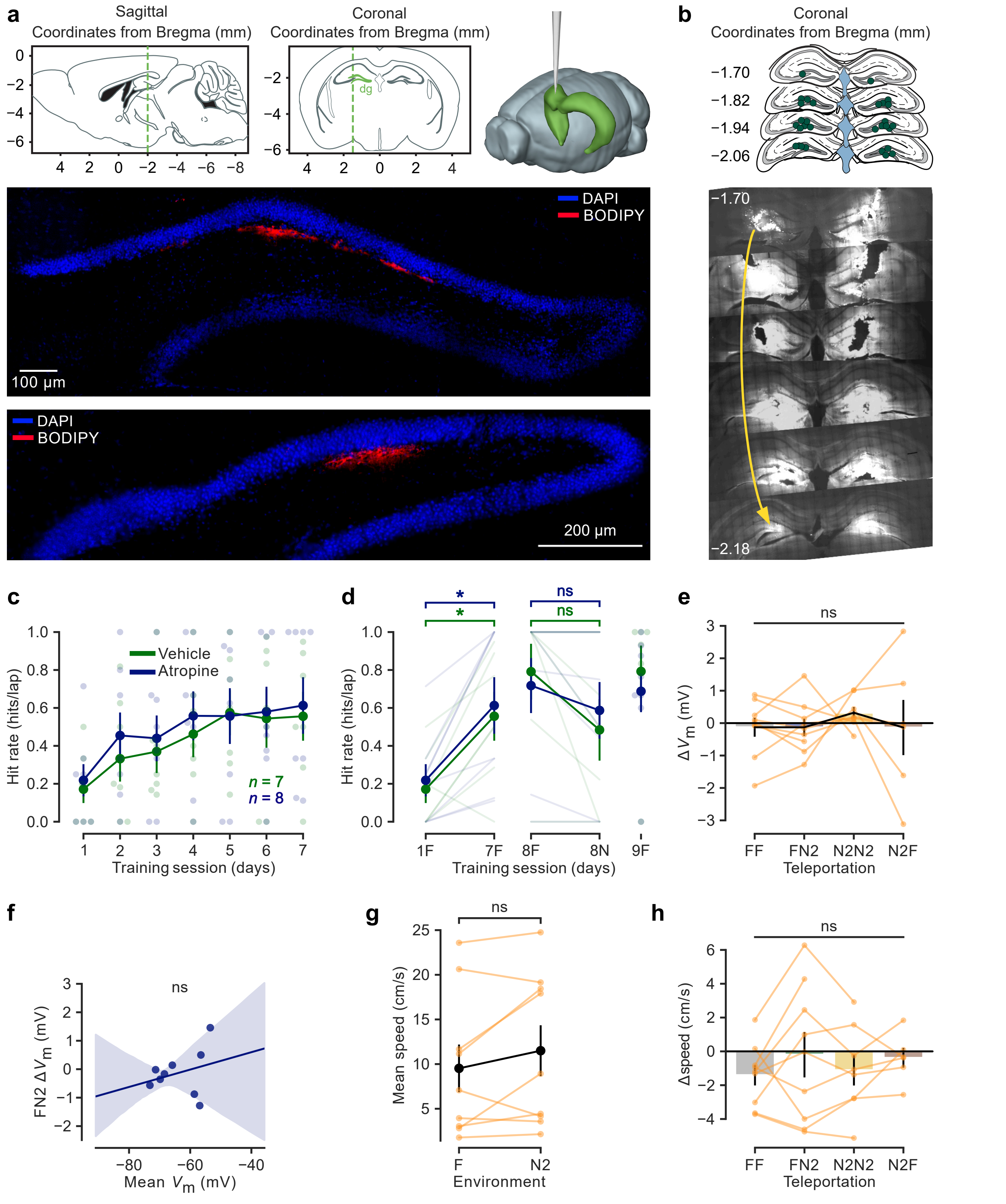

Supplementary Fig. 2 Supplementary analysis to Fig. 4. **a** Stereotaxic injections targeting the dentate gyrus. Coordinates: from Bregma, anteroposterior −2.0 mm, parasagittal +1.5 mm, depth from cortical surface 1.7 mm. Top left: sagittal view. Top middle: coronal view. Top right: 3D schematic of the target injection site. Bottom: two representative examples (from 15 independent experiments with similar results) of an injection of the fluorescent marker BODIPY (red) selectively targeting the upper blade of the dentate gyrus. DAPI (blue) was used as a nuclear stain to reveal the general anatomy of the preparation. Images generated using the Allen Institute Brain Explorer 2 software (`http://mouse.brain-map.org/static/brainexplorer`). The mouse brain drawing is reproduced with permission from The Mouse Brain in Stereotaxic Coordinates, K. B. J. Franklin and G. Paxinos, Figure 48, Copyright Elsevier (2007). **b** Top: summary of cannulae tip positions. Bottom: example coronal section series showing fluorescence in the dentate gyrus after BODIPY infusion through the cannulae (see Methods). **c** behavioural performance across training sessions in animals implanted with chronic cannulae in the dentate gyrus subsequently infused with either vehicle or atropine during the behavioural discrimination session (see Methods). Circles represent individual animals. Bold circles with error bars represent the mean ± s.e.m. across all animals in a group (vehicle, *n* = 7 mice; atropine, *n* = 8 mice). [Legend continues on next page]

Supplementary Fig. 2 Supplementary analysis to Fig. 4. [Continued] **d** Left: behavioural performance during the first (1F) and the last (7F) training sessions in the familiar environment in animals implanted with chronic cannulae in the dentate gyrus subsequently infused with either vehicle (hit rate: 0.17 ± 0.07 hits/lap in 1F and 0.56 ± 0.13 hits/lap in 7F, n = 7 mice, two-sided Wilcoxon signed-rank test, T = 1.0, p = 0.03) or atropine (hit rate: 0.22 ± 0.09 hits/lap in 1F and 0.61 ± 0.15 hits/lap in 7F, n = 8 mice, two-sided Wilcoxon signed-rank test, T = 0.0, p = 0.01) during the behavioural discrimination session. Middle: behavioural performance in the familiar (8F) and in the novel environment 1 (8N) during the 8th training session after local infusion of either vehicle (hit rate: 0.79 ± 0.15 hits/lap in 8F and 0.48 ± 0.16 hits/lap in 8N, n = 7 mice, two-sided Wilcoxon signed-rank test, T = 0.0, p = 0.07) or atropine (hit rate: 0.72 ± 0.15 hits/lap in 8F and 0.59 ± 0.15 hits/lap in 8N, n = 8 mice, two-sided Wilcoxon signed-rank test, T = 0.0, p = 0.07). Right: behavioural performance on a 'wash' training session in the familiar environment (9F) on the day following the behavioural discrimination experiment for the vehicle (hit rate: 0.79 ± 0.69 hits/lap, n = 7 mice,) and the atropine (hit rate: 0.69 ± 0.11 hits/lap, n = 8 mice) groups. **e** ΔV_{m} summary for all teleportations during the behavioural discrimination experiment after local injection of atropine ($-$0.13 ± 0.29 mV for FF, $-$0.13 ± 0.27 mV for FN2, 0.32 ± 0.17 mV for N2N2, $-$0.14 ± 0.85 mV for N2F; n = 9 cells; Friedman test, df = 3, Q = 4.6, p = 0.2). **f** Correlation between Mean V_{m} and FN2 ΔV_{m} during the behavioural discrimination experiment after local injection of atropine (n = 9 cells, Pearson's correlation coefficient, r = 0.28, p = 0.5). **g** Summary of mean speed for the familiar (F) and the novel (N2) environments during the behavioural discrimination experiment after local injection of atropine (9.5 ± 2.7 cm/s and 11.5 ± 2.8 cm/s, respectively; n = 9 cells, two-sided Wilcoxon signed-rank test, T = 12.0, p = 0.2). **h** Δspeed summary for all teleportations during the behavioural discrimination experiment after local injection of atropine ($-$1.4 ± 0.6 cm/s for FF, $-$ 0.2 ± 1.3 cm/s for FN2, $-$1.1 ± 0.9 cm/s for N2N2, $-$0.4 ± 0.6 cm/s for N2F; n = 9 cells, Friedman test, df = 3, Q = 6.7, p = 0.08). V_{m}, membrane potential. Data are presented as the mean ± s.e.m.. Source data are provided as a Source Data file.

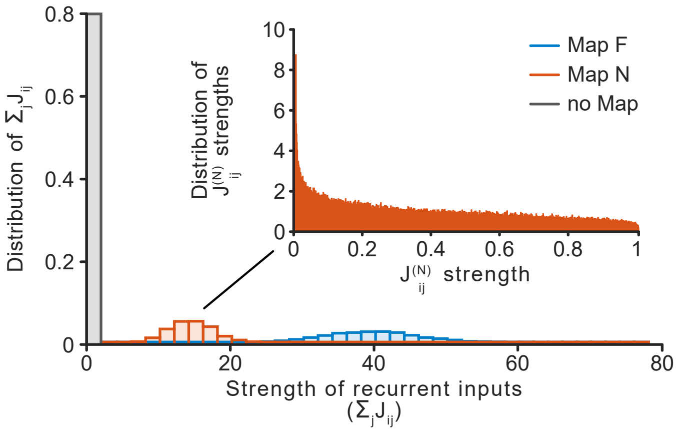

Supplementary Fig. 3 Distribution of synaptic weights in the CA3 network model shows separate bumps for the two maps. Histograms of the summed synaptic inputs for units in map F (blue), units in pre-map N (red) and for the remaining units (grey). Distributions are calculated separately for the two maps with a fraction of units per map of $0.2n$. Sparser and weaker connections for pre-map N result in a lower average synaptic input to its units compared to the subnetwork storing the F map. Insert plot: histogram (normalised as a probability density function) for modified weights for connections in the N map following a beta distribution with parameters α = 0.7, β = 1.2. Map N is initially generated as a consolidated map similarly to map F, non-zero weights are then replaced by the beta distributed random variable in the plot. Notice the peak of the distribution at $J_{ij}^{(N)}$ = 0.

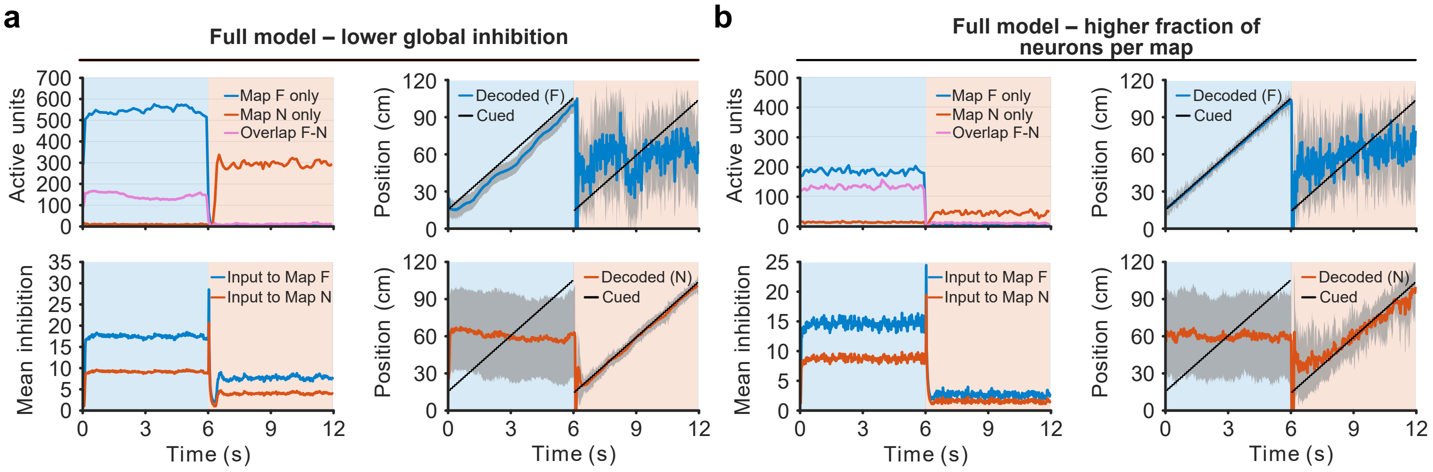

Supplementary Fig. 4 Simulations with alternative sets of network parameters. Effect of global inhibition and fraction of units per map on the network dynamics, represented as in Fig. 5b, 5d, 5e. **a** Model with lower coefficients of global inhibition. Activity (top left) is higher in both maps compared to the model in Fig. 5b with no significant differences in network performance. The stronger bump in map F is associated with stronger recurrent inputs, changing the balance with mEC inputs and effectively slowing the bump from following the animal position in the familiar environment (top right). To balance the difference in inhibition in map N, the threshold of initial activation of the map, $S_{\min}$, has to be lowered accordingly. This new threshold, also affecting map F, requires a corresponding decrease of the coefficient of global inhibition $g_{i,\mathrm{F}}$ also for this map. Finally, the strength of the transient feed-forward inhibition needs to be adapted to the new levels of inhibition in the network to ensure the disruption of the activity bump in map F. The parameters for this simulation, modified from Fig. 5, were $g_{i,\mathrm{N}}$ = 0.01, $g_{i,\mathrm{F}}$ = 0.025, $S_{\min}$ = 80 and $A^{\text{DG-inhib}} = 0.021 \times N_{\text{inter}}$. Similar but opposite variations were also tested (i.e., $g_{i,\mathrm{N}}$ = 0.02, $g_{i,\mathrm{F}}$ = 0.05, $S_{\min}$ = 20 and $A^{\text{DG-inhib}} = 0.012 \times N_{\text{inter}}$) with, again, no significant differences in network dynamics. **b** Model with a higher fraction (0.4n) of units per map. The increased map size with a fixed number of units per activity pattern generates less correlated memories in the network. Lower correlations between the activity patterns require stronger inhibition and mEC inputs for map F to drive its activity bump, resulting in a lower activity in the F map (top left, blue line) compared to Fig. 5b, 5d, 5e. Activity in the F-N overlap units (top left, purple line) is instead compensated by the increased number of units shared by the two subnetworks. The higher inhibition for units in the F map together with the higher overlap result in a reduced activity (top left, red line) and a noisier spatial selectivity (bottom right) for the N map. The parameters modified from Fig. 5 were $g_{i,\mathrm{F}}$ = 0.045, $S_{\min}$ = 45, $A^{\text{mEC,F}}$ = 5 and $A^{\text{DG-inhib}} = 0.021 \times N_{\text{inter}}$. Grey shaded areas represent the standard deviation associated with the decoded position.

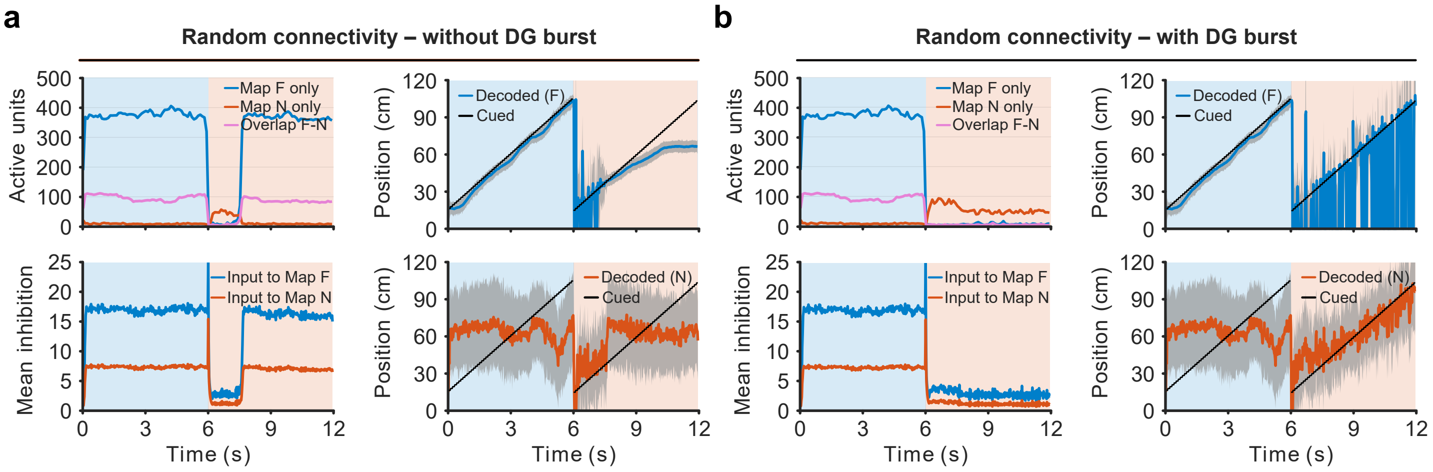

Supplementary Fig. 5 Simulations with random connectivity in sub-network N. Simulations without a spatially-structured pre-wired connectivity between the units supporting the (to be formed) novel map. Network dynamics do not differ significantly from the results shown in Fig. 5b and 5d for a network with a structured pre-wired connectivity encoding the novel environment. **a** Model without the transient input from DG, as in Fig. 5d. After teleportation, the initial switch to the ensemble of units which, after a learning phase, will encode the novel environment is short-lived. Without the support of the transient increased DG input, activity is immediately reinstated in the consolidated F map. Decoded position in both maps (right panels) is calculated as the mean position of the activity bump. The higher variability in the spatial tuning of active units in the N environment, due to the lack of structure of synaptic connections, resulted in less accurate decoded positions compared to previous simulations. To account for this effect, only place fields supported by at least 2 active units were included in the evaluation of the decoded position. Due to this threshold, decoded position using the F map for the N environment results in a discontinued line because of the very low number of active units in the F sub-network. Nonetheless, the few active units in the F sub-network have a place field close to the rodent's position in the N environment, due to the space-selective mEC input. **b** Complete model, including the transient increased input from DG after teleportation, as in Fig. 5b. The novel cell assembly is activated after the switch of environment as in the pre-wired case but with slightly lower total activity in the network. As for panel **a**, activity was thresholded to evaluate the decoded position which shows, for environment N (bottom right), a small but stable bump of activity following the rodent's position as cued from mEC. Note the very weak activity of neurons coding for the F map while in the N environment (top left), mostly due to mEC cues, which occasionally allows for decoding of position in this map (top right). Grey shaded areas represent the standard deviation associated with the decoded position.

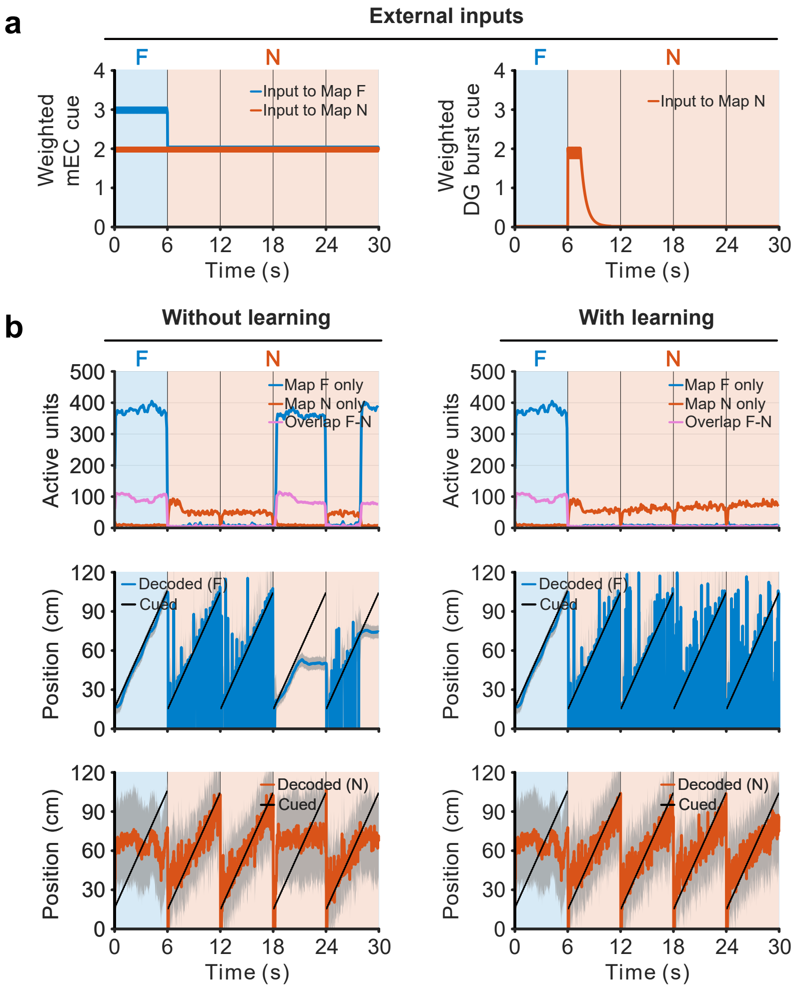

Supplementary Fig. 6 Effect of Hebbian learning with a random connectivity in sub-network N. Synaptic plasticity supports the formation of a new representation for the novel environment while maintaining and stabilising the activity of the new cell assembly even when DG activity returns to its baseline level. Simulations were performed with an initially random and unstructured pre-wired synaptic connectivity for map N. **a** mEC (left) and DG (right) inputs, as in Fig. 5b, for both sets of simulations in panel **b**. The virtual rodent, after a first exploration of environment F, is teleported to environment N for four consecutive exploration laps. mEC inputs remain constant while in map N (i.e., mEC-CA3 synaptic connections are not strengthened) while DG inputs transiently increase from baseline level only after the first teleportation to the novel environment. Black vertical lines indicate a teleportation event. **b** Simulations with (right, η = 0.1) and without hebbian learning (left, η = 0). After the first teleportation to the novel environment, the CA3 network cannot rely neither on the increased DG input nor on a prevailing mEC cue to activate the proper cell assembly, given the current map. Therefore, as shown in left figures, network activity is unstable and jumps between the two available sub-networks. Conversely, when hebbian learning is introduced during the first exploration laps in the N environment, the N sub-network is consistently activated and reinforced, as shown by its slowly increasing number of active units (top right, red line). Grey shaded areas represent the standard deviation associated with the decoded position.
