# Supplementary material for: A synaptic signal for novelty processing in the hippocampus
Source: Nat Commun. 2022 Jul 15;13:4122. doi: 10.1038/s41467-022-31775-6 (PMC9287442; doi:10.1038/s41467-022-31775-6)
Supplement: Supplementary file 2 — Description of Additional Supplementary Files [file 41467_2022_31775_MOESM2_ESM.pdf]

Description of Additional Supplementary Files

Supplementary Movie 1: Network activity during two simulated lap crossings across the familiar (F) and the novel (N) environment. Blue and red bars represent the number of active cells with a place field centre in the corresponding 5 cm bin in, respectively, the F (top) and N (middle) maps. Black dashed lines represent the position cued through the mEC input to the subnetworks involved in the encoding of the two maps. Simulated animal position is shown at the bottom. Note the activity bump moving from the F to the N map after teleportation**.**

Supplementary Code: MATLAB scripts developed to implement the attractor neural network and its dynamics. The code for the two models presented in the main text and in the Supplementary Information can be found in the respective folders. Folder *F2N_StructuredPremap* contains the code for the network model with random but structured connectivity supporting the novel map representation (N). Folder *F2N_RandomPremap_Learning* contains the code for the network model with random and unstructured connectivity supporting the N map. Additionally, in this script is implemented the possibility to simulate the Hebbian learning of the novel environment.
