# Supplementary material for: A synaptic signal for novelty processing in the hippocampus
Source: Nat Commun. 2022 Jul 15;13:4122. doi: 10.1038/s41467-022-31775-6 (PMC9287442; doi:10.1038/s41467-022-31775-6)
Supplement: Supplementary file 5 — Reporting Summary [file 41467_2022_31775_MOESM5_ESM.pdf]

nature research

Corresponding author(s): Christoph Schmidt-Hieber

Last updated by author(s): May 31, 2022

# Reporting Summary

Nature Research wishes to improve the reproducibility of the work that we publish. This form provides structure for consistency and transparency in reporting. For further information on Nature Research policies, see our Editorial Policies and the Editorial Policy Checklist.

## Statistics

For all statistical analyses, confirm that the following items are present in the figure legend, table legend, main text, or Methods section.

| n/a | Confirmed | |
|---|---|---|
| ☐ | ☒ | The exact sample size ($n$) for each experimental group/condition, given as a discrete number and unit of measurement |
| ☐ | ☒ | A statement on whether measurements were taken from distinct samples or whether the same sample was measured repeatedly |
| ☐ | ☒ | The statistical test(s) used AND whether they are one- or two-sided<br>*Only common tests should be described solely by name; describe more complex techniques in the Methods section.* |
| ☐ | ☒ | A description of all covariates tested |
| ☐ | ☒ | A description of any assumptions or corrections, such as tests of normality and adjustment for multiple comparisons |
| ☐ | ☒ | A full description of the statistical parameters including central tendency (e.g. means) or other basic estimates (e.g. regression coefficient) AND variation (e.g. standard deviation) or associated estimates of uncertainty (e.g. confidence intervals) |
| ☐ | ☒ | For null hypothesis testing, the test statistic (e.g. $F$, $t$, $r$) with confidence intervals, effect sizes, degrees of freedom and $P$ value noted<br>*Give P values as exact values whenever suitable.* |
| ☒ | ☐ | For Bayesian analysis, information on the choice of priors and Markov chain Monte Carlo settings |
| ☒ | ☐ | For hierarchical and complex designs, identification of the appropriate level for tests and full reporting of outcomes |
| ☐ | ☒ | Estimates of effect sizes (e.g. Cohen's $d$, Pearson's $r$), indicating how they were calculated |

*Our web collection on statistics for biologists contains articles on many of the points above.*

## Software and code

Policy information about availability of computer code

| | |
|---|---|
| Data collection | Behaviour and electrophysiology data were acquired with the Blender Game Engine (http://www.blender.org; v3.1.2) in conjunction with custom Python scripts (https://www.python.org/; v3.10.4) using the Blender Python Application Programming Interface (http://www.blender.org; v3.1.2). |
| Data analysis | Behaviour and electrophysiology data were analysed using custom Python scripts (https://www.python.org/; v3.10.4). Fluorescence images were analysed using ImageJ (https://imagej.nih.gov/ij/; v1.8.0_172). Simulations were implemented using custom MATLAB scripts (https://se.mathworks.com/products/matlab.html; vR2017b/2020b), which are provided with this paper and its Supplementary Information files. |

For manuscripts utilizing custom algorithms or software that are central to the research but not yet described in published literature, software must be made available to editors and reviewers. We strongly encourage code deposition in a community repository (e.g. GitHub). See the Nature Research guidelines for submitting code & software for further information.

## Data

Policy information about availability of data

All manuscripts must include a data availability statement. This statement should provide the following information, where applicable:

- Accession codes, unique identifiers, or web links for publicly available datasets
- A list of figures that have associated raw data
- A description of any restrictions on data availability

A Source Data file supporting the findings of this study is provided with this paper and its Supplementary Information files. Additional binary data files are available from the corresponding author upon request.

# Field-specific reporting

Please select the one below that is the best fit for your research. If you are not sure, read the appropriate sections before making your selection.

☒ Life sciences ☐ Behavioural & social sciences ☐ Ecological, evolutionary & environmental sciences

For a reference copy of the document with all sections, see nature.com/documents/nr-reporting-summary-flat.pdf

# Life sciences study design

All studies must disclose on these points even when the disclosure is negative.

| | |
|---|---|
| Sample size | No sample-size calculation was performed. Sample sizes were chosen based on similar published studies using comparable approaches (Schmidt-Hieber and Häusser, 2013, DOI: 10.1038/nn.3340; Allegra et al., 2022, DOI: https://doi.org/10.1016/j.neuron.2020.09.032). |
| Data exclusions | Data were excluded from analysis according to criteria defined a priori to ensure the quality and completeness of the behavioural and electrophysiological recordings. Electrophysiology data were excluded from analysis if the membrane seal resistance was < 1GΩ, if the recorded cell failed to produce action potentials in response to current injection pulses, if the quality of the recording was lost before acquisition of sufficient behavioural data (increase in series resistance or depolarisation requiring injection of a holding current to preserve the baseline membrane potential) or if obvious electrostatic artefacts were present in the traces. Behavioural data were excluded from analysis if a recording session did not include sufficient sampling of both virtual environments. |
| Replication | All experimental findings were reproducible in recordings meeting the pre-established inclusion and exclusion criteria. The number of independent replicates for each experiment is indicated in the manuscript. |
| Randomization | Allocation of animals into experimental groups was not randomised. All animals were treated identically. |
| Blinding | Investigators were blinded to experimental group allocation during data collection in behavioural experiments comparing local infusion of vehicle vs atropine in the dentate gyrus (drug infusion and behavioural recordings performed by different investigators). Investigators were not blinded to experimental group allocation during data collection or analysis in electrophysiology experiments due to limitations of the experimental design (injection of drug and immediate electrophysiological recordings performed as a block by a single investigator). |

# Reporting for specific materials, systems and methods

We require information from authors about some types of materials, experimental systems and methods used in many studies. Here, indicate whether each material, system or method listed is relevant to your study. If you are not sure if a list item applies to your research, read the appropriate section before selecting a response.

## Materials & experimental systems

| n/a | Involved in the study | |
|---|---|---|
| ☒ | ☐ | Antibodies |
| ☒ | ☐ | Eukaryotic cell lines |
| ☒ | ☐ | Palaeontology and archaeology |
| ☐ | ☒ | Animals and other organisms |
| ☒ | ☐ | Human research participants |
| ☒ | ☐ | Clinical data |
| ☒ | ☐ | Dual use research of concern |

## Methods

| n/a | Involved in the study | |
|---|---|---|
| ☒ | ☐ | ChIP-seq |
| ☒ | ☐ | Flow cytometry |
| ☒ | ☐ | MRI-based neuroimaging |

## Animals and other organisms

Policy information about studies involving animals; ARRIVE guidelines recommended for reporting animal research

| | |
|---|---|
| Laboratory animals | All experiments included in this study were performed in 5-16 week-old male C57BL/6J wild-type mice (Janvier Labs). Animals were housed in groups of four in polycarbonate individually-ventilated cages equipped with running wheels and were kept under constant temperature and humidity with a 12-h inverted light/dark cycle and ad libitum access to food and water. All animals were treated identically. |
| Wild animals | This study did not involve wild animals. |
| Field-collected samples | This study did not involve samples collected from the field. |
| Ethics oversight | All procedures in this study were conducted in accordance with European and French regulations on the ethical use of laboratory animals for experimentation (EU Directive 2010/63/EU) and were reviewed and approved by the Ethics Committee of the Institut Pasteur CETEA (APAFIS#7771-2016112516084126 v1). |

Note that full information on the approval of the study protocol must also be provided in the manuscript.
